# Activation of the circAGFG1/miR-195-5p/PD-L1 axis induces lung injury in sepsis

**DOI:** 10.1007/s13577-025-01258-z

**Published:** 2025-07-14

**Authors:** Yang Bi, Rui Ding, Xinyan Liu, Yun He, Xukun Liu, Shouye Ma, Chaofan Wang, Zhongfa Zhang, Xuan Song

**Affiliations:** 1 Shandong First Medical University, Jinan, 250117 Shandong China; 2 The Second School of Clinical Medical, Binzhou Medical University, Yantan, 264000 Shandong China; 3 ICU, Dong E Hospital, Liaocheng, 252200 Shandong China; 4 Shandong Provincial Clinical Research Center for Infectious Diseases, Public Health Clinical Center Affiliated to Shandong University, Shandong Public Health Clinical Center, Jinan, 250102 Shandong China; 5 School of Clinical and Basic Medicine, Shandong First Medical University and Shandong Academy of Medical Sciences, Jinan, 250117 Shandong China

**Keywords:** Sepsis, Acute lung injury, circAGFG1, miR-195-5p, PD-L1

## Abstract

Severe sepsis can escalate to acute lung injury (ALI), a critical condition with limited treatment options. Emerging research highlights the role of non-coding RNAs, including microRNAs (miRNAs) and circular RNAs (circRNAs), in regulating epithelial and immune cell responses in sepsis-induced ALI. This study delves into the circAGFG1/miR-195-5p/PD-L1 axis to elucidate its potential in this context. The conducted experiments using Th17 cells differentiated from CD4+ T cells isolated from human umbilical cord blood, co-cultured with transfected Calu-3 cells. The assessed cell viability, RNA, and protein expression levels under inflammatory stimuli. In addition, we collected bronchoalveolar lavage fluid and lung tissues from a cecal ligation and puncture mouse model. Quantification of RNA and protein expression of inflammatory and survival markers provided insights into the regulatory mechanisms involved. Peripheral blood samples from sepsis/ALI patients and healthy controls revealed that circAGFG1 expression is significantly downregulated, while miR-195-5p expression is upregulated in patients compared to healthy individuals. In both Calu-3 cells and induced Th17 cells, as well as in sepsis-induced ALI mouse models, the circAGFG1/miR-195-5p/PD-L1 axis was shown to regulate the expression of inflammatory cytokines, Th17 cell differentiation, and markers of epithelial cell survival. Results show the circAGFG1/miR-195-5p/PD-L1 axis plays a critical role in the pathogenesis of sepsis-induced ALI. Targeting this axis could present a novel therapeutic strategy for preventing ALI in sepsis and other conditions characterized by heightened lung inflammation. This research paves the way for new treatment approaches, offering hope for improved outcomes in patients suffering from severe sepsis and ALI.

**Graphical abstract:**

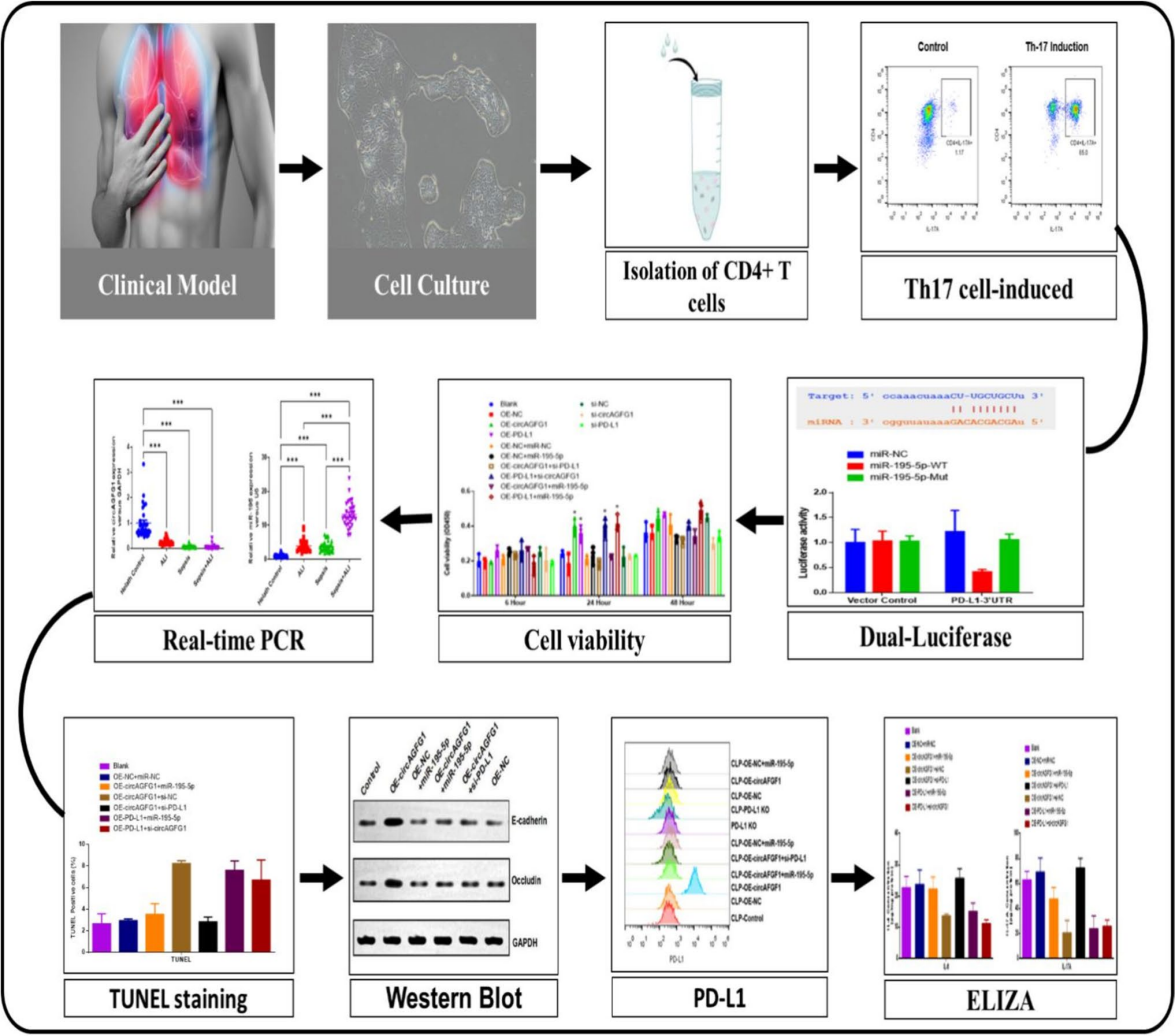

**Supplementary Information:**

The online version contains supplementary material available at 10.1007/s13577-025-01258-z.

## Introduction

Sepsis is a systemic inflammatory response induced by microbial infection, causes severe illnesses and is associated with high mortality rates [1, 2]. Studies have shown that about 48.9 million sepsis cases and 11 million deaths occur annually worldwide, with the incidence rate expected to rise [3–5]. Half of patients with severe sepsis develop either acute lung injury (ALI) or acute respiratory distress syndrome (ARDS), which can lead to multiple organ failure, with a 40% mortality rate [6–8]. Current treatment options for sepsis-induced ALI/ARDS include lung-protective mechanical ventilation, extracorporeal membrane oxygenation, conservative fluid management, neuromuscular blocking agents, corticosteroids, and inhaled nitric oxide. However, these treatment modalities have low efficacy, and mortality rates have remained high [9].

Elucidating the mechanism underlying the pathogenesis of sepsis-induced ALI can provide novel molecular targets, thus setting up a theoretical basis of guide development of prevention and treatment therapies. Pulmonary epithelial cell injury due to infection-induced senescence, apoptosis, and inflammation is a major cause of sepsis-induced ALI [9]. Its pathogenesis is regulated by the programmed death-1 (PD-1)/programmed death ligand-1 (PD-L1) pathway, which inhibits T cell response and downregulates expression of anti-apoptotic molecules as well as inflammatory factors [10–15]. Studies have shown that PD-L1 is widely expressed on the surface of both airway epithelial and pulmonary endothelial cells [16]. This phenomenon causes over-activation of the PD-1/PD-L1 pathway and upregulates inflammatory cytokines thereby inducing sepsis [17, 18]. Results from an ALI study demonstrated that patients who died were associated with higher PD-1 levels than survivors [19]. Although additional studies have demonstrated the role of PD-1 in sepsis-induced ALI, little is known regarding the regulation of PD-L1 expression in airway epithelial cells and the mechanism underlying immune evasion during ALI/ARDS [20].

Non-coding RNAs, such as long non-coding RNAs (lncRNA), micro RNAs (miRNA), and circular RNAs (circRNA), are a current research hotspot, owing to their implications in several cellular processes [12, 21–24], including sepsis-induced ALI. In ALI mice, miR-155 levels in peripheral blood were associated with increased number of lung macrophages [25], while over-expression of miR-454 suppressed inflammatory cytokines, including tumor necrosis factor-α (TNF-α) [26]. Other miRNAs involved in inflammation regulation include miRNA-29a [27], miRNA-127 [28], and miR-195-5p, which participates in sepsis-induced ALI [29]. Studies have also described miRNA-mediated regulation of PD-L1 expression in solid tumors, leukemia, and other cancers [30–36], including regulation by miR-195-5p [37–40]. Combined with our previous work [30] findings, these evidences suggest that miR-195-5p may target PD-L1 expression, thereby affecting airway epithelial cell resistance to ALI. Studies have demonstrated that circRNAs may regulate miRNA function via the competitive endogenous RNA mechanism thus preventing them from binding their targets [41–43]. For instance, circAGFG1 regulates several miRNAs, including miR-28-5p and miR-203 [44, 45], miR-4306 [46], and miR-370-3p [47]. To date, however, it remains unclear whether circAGFG1 regulates the miR-195-5p/PD-L1 pathway in sepsis-induced ALI.

In the present study, we hypothesized that high circAGFG1 expression in airway epithelial cells could inhibit miR-195-5p in sepsis-induced ALI, thereby improving airway epithelial cell proliferation and inhibiting Th17 cell function via upregulation of PD-L1 expression. Therefore, we analyzed expression patterns of circAGFG1, miR-195-5p, and PD-L1 in sepsis-induced ALI patients to elucidate the mechanism underlying regulation of the circAGFG1/miR-195-5p/PD-L1 axis in airway epithelial cells and a sepsis-induced ALI murine model.

## Materials and methods

### Clinical model

We enrolled patients with sepsis, ALI, as well as those with sepsis complicated with ALI who were treated at our hospital. All patients met the international criteria for diagnosis and treatment of sepsis and septic shock, according to the guidelines established in 2016 [48], and the diagnostic criteria of ALI and respiratory distress syndrome, according to the guidelines established in 2006 [49]. The clinical characteristics of patients are presented in Table 1. Each patient’s peripheral blood was collected, under aseptic conditions, with blood from healthy subjects also obtained as control. This study has been approved by the Ethics Review Committee of Shandong First Medical University (Approval No.: R202103030020), and all patients voluntarily signed a written informed consent prior to inclusion in the study.

**Table 1:** Clinical characteristics of patients in this study

	Sepsis (*n* = 30)	ALI (*n* = 30)	Sepsis with ALI (*n* = 30)	*Χ*^2^/*t*	*P*
Male sex	20	23	22	1.587	0.662
Age, years	65.90 ± 13.33	60.50 ± 16.74	65.77 ± 7.06	1.451	0.232
Chronic comorbidity, n (%)					
None	6 (20.0%)	3 (10.0%)	5 (16.7%)	1.184	0.553
Cardiovascular disease	7 (23.3%)	15 (50.0%)	9 (30.%)	5.118	0.077
Chronic pulmonary disease	3 (10.0%)	6 (20.0%)	4 (13.3%)	1.259	0.533
Diabetes mellitus	4 (13.3%)	9 (30.0%)	9 (30.0%)	3.008	0.222
Cerebrovascular disease	10 (33.3%)	15 (50.0%)	8 (26.7%)	3.732	0.155
Immune system disease	0	2 (6.7%)	0	4.091	0.129
Chronic liver disease	0	2 (6.7%)	1 (3.3%)	2.069	0.355
Chronic kidney disease	1 (3.3%)	2 (6.7%)	3 (10.0%)	1.071	0.585
Tumor	6 (20.0%)	1 (3.3%)	1 (3.3%)	6.707	0.035
Severity of disease on admission					
APACHE II score, mean (SD)	17.63 ± 6.39	25.90 ± 8.23	20.00 ± 7.19	10.174	< 0.001
SOFA score, mean (SD)	5.37 ± 2.40	8.67 ± 4.26	6.00 ± 2.91	8.519	< 0.001
Outcomes					
ICU length of stay, days	8.77 ± 8.55	7.43 ± 4.61	10.63 ± 6.43	1.713	0.186
Hospital length of stay, days	22.93 ± 25.68	12.27 ± 6.77	17.37 ± 10.75	3.120	0.049
28-day mortality, n (%)	8 (26.7%)	10 (33.3%)	16 (53.3%)	4.916	0.086

The expression profiles of circRNA and miRNA in peripheral blood were detected by microarray analysis. Briefly, the total RNAs were subjected to digestion with RNase R (RNR07250, Epicentre) to eliminate linear RNAs and enrich circular RNAs. The enriched circular RNAs were then amplified and transcribed into fluorescent cRNA using a random priming method provided by the Arraystar Super RNA Labeling Kit. The labeled cRNAs were subsequently hybridized onto the Arraystar Human circRNA Array V2 (8 × 15 K, Arraystar). Following slide washing, the arrays were scanned using the Agilent Scanner G2505C. Analysis of acquired array images was performed using the Agilent Feature Extraction software (version 11.0.1.1). Differentially expressed circRNAs (fold change [FC] ≥ 2 and *p* < 0.05) were identified by VolcanoPlot filtering, while levels of circAGFG1 and miR-195-5p expression in peripheral blood were analyzed via quantitative real-time polymerase chain reaction qRT-PCR as described above.

### Cell cultures

Human non-small-cell lung cancer cell line Calu-3 was obtained from the American Type Culture Collection (ATCC). The cells were cultured in T75 flasks with minimum essential medium (Gibco, Carlsbad, CA, USA), supplemented with heat-inactivated 10% fetal bovine serum (FBS, Sigma-Aldrich), 1% L-glutamine, 1% sodium pyruvate, and 1% penicillin/streptomycin. The cultures were grown in an incubator with CO_2_ concentration of 5% and a temperature of 37℃.

### Isolation of CD4+ T cells

To isolate human peripheral blood CD4+ T cells, collect peripheral blood samples from healthy human donors using standard venipuncture techniques with EDTA as an anticoagulant. Dilute the blood sample with an equal volume of PBS and layer it onto a density gradient medium. Centrifuge at a low speed for 30 min to separate peripheral blood mononuclear cells (PBMCs). Collect the PBMC layer, wash with PBS, and resuspend in PBS with 2% FBS. Isolate CD4+ T cells using negative selection methods based on human CD4+ T Cell Isolation Kit (Thermo Fisher, USA). Wash the isolated CD4+ T cells and assess cell viability. Th17 cells were differentiated from CD4+ T cells isolated from human umbilical cord blood. Human umbilical cord blood mononuclear cells (UCBMCs) were isolated from umbilical cord blood donated by healthy pregnant women. Blood was first centrifuged for 10 min to remove plasma, the supernatant diluted with phosphate-buffered saline (PBS), and UCBMCs were obtained by density gradient centrifugation. CD4+ T cells were isolated by LS column.

### Th17 cell-induced differentiation

10^4^ CD4+ T cells were first isolated as described above, then incubated for 24 h in CD3-coated (5 μg/mL) 96-well plates (Thermo Fisher, USA) supplemented with soluble CD28 (2 μg/ml). Differentiation was induced after 24 h of incubation, by adding 20 μg/mL IL-6(R&D Systems, USA), 5 μg/mL transforming growth factor (TGF-β)(R&D Systems, USA), 10 μg/mL IL-23(R&D Systems, USA), 2 μg/mL anti-IL-4(Abcam, USA), and 2 μg/mL anti-INF-γ(Abcam, USA), followed by a 3-day incubation. The culture medium was replaced once each day. On the third day, cell clumping in U-shaped bottom 96-well plates was analyzed under a microscope, which revealed obvious cell precipitation at the bottom of the well. The proportion of Th17 cells was analyzed via flow cytometry on the third day following induction.

### Flow cytometric analysis of Th17 differentiation

Samples were mixed with a leukocyte activation cocktail with GolgiPlug (2 μg/ml, BD Biosciences) and incubated in a 5% CO_2_ incubator at 37℃ for 4 h. The cells were collected, transferred to a flow tube, and centrifuged at 300 rpm for 5 min. The supernatant was removed, and the cells resuspended in 100 μL of flow buffer. The sample was then incubated with a PE-CD4 antibody (1 μL) (Biolegend, USA) at room temperature for 30 min, washed once, and permeabilized with 250 μL 4% Paraformaldehyde (PFA) solution. Next, the sample was incubated at 4℃ in the dark for 40 min, and rinsed twice with permeabilization. The cells were resuspended in 50 μL of fixative, incubated at room temperature with 1 μL of FITC-IL-17A antibody (Biolegend, USA) for 40 min, washed once with flow buffer, and resuspended in 300 μL PBS. Finally, flow cytometry was employed to quantify levels of CD4 and IL-17 expression. The gating strategy was determined based on isotype controls. Data were acquired on a FACSCalibur (BD Biosciences, USA) and analyzed using FlowJo software (Treestar).

### Cell transfection

Calu-3 cells were first seeded in a 6-well plate at a density of 2 × 10^4^ cells per well, and incubated for 24 h.

To transfect miRNA and plasmid, we utilized a Lipofectamine 3000-based transfection method. Maintain the Calu-3 cells in appropriate growth media under standard culture conditions until they reach approximately 70–80% confluence. In two separate tubes, prepare transfection complexes for miRNA 195 mimic/inhibitor and PD-L1 plasmid. For each tube, dilute the desired amount of miRNA 195 mimic/inhibitor and PD-L1 plasmid in Opti-MEM or serum-free medium as recommended by the transfection reagent manufacturer. Add an equal volume of transfection reagent Lipofectamine 3000 to each tube containing the diluted miRNA or plasmid DNA. Mix gently and incubate the transfection complexes at room temperature for 20 min to allow for complex formation. After the incubation period, add the transfection mixture containing the miRNA 195 mimic/inhibitor and PD-L1 plasmid to the respective wells of the cell culture plate. Mix gently by swirling the plate. Place the cell culture plate back into the cell incubator and allow the cells to incubate for 24 h, to allow for efficient transfection and expression. The cells were collected via centrifugation 24 h after transfection. PD-L1/circAGFG1 over-expression plasmids and siRNAs were transfected according to the groups, and cells were harvested 24 h later. The relevant primer sequences are shown in Table 2.

**Table 2:** Primer sequence

	Sense (5'–3')	Antisense (5'–3')
has-miR-195-5p	UAGCAGCACAGAAAUAUUGGC	CAAUAUUUCUGUGCUGCUAUU
hsa_circ_0058514-1	GUCAUGCAGGCGAGGAUUAAATT	UUUAAUCCUCGCCUGCAUGACTT
hsa_circ_0058514-2	CAGUCAUGCAGGCGAGGAUUATT	UAAUCCUCGCCUGCAUGACUGTT
hsa_circ_0058514-3	CAACAGUCAUGCAGGCGAGGATT	UCCUCGCCUGCAUGACUGUUGTT
mmu-miR-195-5p	UAGCAGCACAGAAAUAUUGGC	CAAUAUUUCUGUGCUGCUAUU
Mut-miR-195-5p	UUCGUCGCUCUAAAAAUGGC	CAUUUUUAGAGCGACGAAUU
hPD-L1-442	CGAAUUACUGUGAAAGUCAAUTT	AUUGACUUUCACAGUAAUUCGTT
hPD-L1-107	GGCAUUUGCUGAACGCAUUUATT	UAAAUGCGUUCAGCAAAUGCCTT
hPD-L1-223	GCACUAAUUGUCUAUUGGGAATT	UUCCCAAUAGACAAUUAGUGCTT

### Dual-luciferase assay

The predicted binding region of circAGFG1 and miRNA and the 3'UTR region of PD-L1 were inserted into psiCHECK-2 luciferin reporter vector, and transfected into the immortalized human embryonic kidney HEK293 cells. The wild-type and binding site mutant miR-195-5p mimics were also constructed. The miR-195-5p wild-type or mutant mimics were co-transfected into the HEK293 cells with psiCheck-circAGFG1/PD-L1-3’UTR vectors, and the expression level of luciferase was detected. Cell lysates were collected 48 h after transfection and Renilla and firefly luciferase activities were measured in relative light units (RLU) using the Dual-Glo Luciferase Assay System (Cat. No. E2920, Promega) and Cytation 5 multi-mode plate reader (Biotek, Winooski, VT). Renilla luciferase activity was normalized to the internal control firefly luciferase activity. Firefly luciferase is used as internal control luciferase for normalization because psiCHECK-2 provides a moderate-level and constitutive expression of firefly luciferase among experimental groups. Relative luciferase activities are ratios of Renilla/Firefly RLU normalized to negative control for each reporter construct.

### Co-culture of Th17 and Calu-3 cells

Th17 cells were induced as described above, then seeded in the lower chamber of a 12-well plate (10^6^ per well) with a 0.4 μm aperture (Corning, Art. No. 3201). Transfected Calu-3 cells were plated at a density of 5 × 10^5^ cells per well and incubated for 24 h. The next day, unmodified Calu-3 cells were added to the lower chamber of the plate with adhered Th17 cells and cultured with Th17 differentiation medium.

### Cell viability assay

Cell viability was evaluated via the Cell Counting Kit 8 (CCK-8) assay. In brief, Calu-3 cells were seeded in 96-well plates at a density of 1,000 cells per well, and cultured for 6 h, then transfected according to the aforementioned groups. Cells were incubated for 24 h after transfection, then incubated for a further 6, 24, or 48 h. Next, 10 μL of CCK-8 solution was added to each well, and the cells incubated for another 1 h at 37 °C. Finally, the optical density (OD) for each well was measured at 450 nm using a microplate reader. All cell proliferation assays were conducted in triplicate.

#### RNA extraction

RNA was extracted using TRIzol (Invitrogen), according to the manufacturer’s instructions. Briefly, 1 mL TRIzol was added to a cell lysate or tissue and the contents incubated at room temperature for 5 min. Next, RNA was extracted by adding 0.2 mL chloroform to the sample, vortexed for 15 s, followed by a 2–3-min incubation at room temperature. The sample was centrifuged at 12,000×*g* at 4℃ for 15 min, the supernatant aspirated out then mixed with 0.5 mL isopropyl alcohol. The sample was incubated at room temperature for 10 min, centrifuged at 12,000×*g* at 4℃ for 10 min, the supernatant discarded, the pellet washed with 1 mL of 75% ethanol and then resuspended in diethylpyrocarbonate (DEPC)-treated water. The sample was centrifuged at 7500×*g* at 4℃ for 5 min, the supernatant was removed, and the sample was dried in air for 5–10 min. Finally, DEPC-treated double-distilled water (ddH_2_O) was added, and the sample was incubated at 55–60℃ for 10 min. RNA concentration and purity were measured using a NanoDrop 2000 (ThermoFisher), while its integrity was checked by agarose electrophoresis.

#### Complementary DNA (cDNA) synthesis

Next, 1 μg of the RNA was mixed with Oligo-dT primers (ThermoFisher) and ddH_2_O and incubated for 5 min at 70℃. The sample was placed on ice, then mixed with 4 μL 5 × reverse transcriptase (RT) Buffer (ReverTra Ace qPCR RT Master Mix, TOYOBO, Osaka, Japan) and 2 μL 10 mM dNTPs (ThermoFisher), followed by a 5-min incubation at 37℃. Finally, 0.5 μL of RT (TOYOBO, Osaka, Japan) was added, and the sample was incubated at 42℃ for 60 min and 70℃ for 5 min.

#### qRT-PCR

The cDNA was diluted 10 × and 4 μL was added to each well of a 384-well plate and centrifuged at 1,000 rpm. qRT-PCR was performed using the SYBR Green PCR master mix (ThermoFisher, 4,368,708), according to the manufacturer’s instructions, the primers (Sangon Biotech, Shanghai, China) for the indicated genes, and water were mixed at a ratio of 1:1:3, added to each well and mixed again, and then qRT-PCR was performed. The Vii7 system (Applied Biosystems) was adopted to perform qRT-PCR in triplicate for determining the expression. GAPDH and U6 were regarded as the endogenous reference gene for the circAGFG1 and PD-L1 and the loading control for the miR-195-5p, respectively. The 2^−ΔΔCT^ method helped to assess the relative expression.

#### TUNEL staining

Calu-3 cells were seeded in 6-well plates at a density of 2 × 10^4^/mL cells per well, and then transfected with specific constructs as described above. Twelve hours after transfection, the cells were treated with LPS (100 ng/mL), and stained 24 h following LPS induction. Next, the cells were washed twice with PBS, fixed with 4% paraformaldehyde at room temperature for 15 min, and then washed three times with PBS (5 min for each wash). Next, the cells were permeated with PBS supplemented with 0.1% Triton-X-100 at room temperature for 15 min, washed three times with PBS, and then incubated for 1 h with 50 μL of TUNEL staining solution at room temperature in the dark. Next, the cells were washed three times with PBS at room temperature (10 min for each wash), then incubated with DAPI staining solution at room temperature for 10 min. The cells were washed again with PBS, three times for 10 min each, wells sealed with an anti-quenching agent, and the contents visualized under a fluorescence microscope. Images were captured using laser confocal microscopy (LSM 800, Zeiss).

#### Western blot assay

Calu-3 cells were first seeded in 6-well plates, at a density of 10^4^/mL cells per well, incubated for 24 h, then transfected with the specific constructs followed by a 12-h incubation. Next, the cells were treated with LPS (100 ng/mL) for 24 h, protein extracted, and its concentration measured via the bicinchoninic acid (BCA) assay. An equal concentration of protein per sample was separated via SDS-PAGE gel electrophoresis, and then transferred onto polyvinylidene difluoride (PVDF) membranes. The membranes were then incubated with the indicated primary antibody, followed by incubation with the secondary antibodies and then subjected to chemiluminescence. The film was scanned, and the gray density of the target bands was analyzed by Quantity One software.

#### Establishment of an animal model

Animal experiments were approved by the Ethics Review Committee of Shandong First Medical University (Approval No.: W202103030032), with all animal experimental procedures performed in accordance with ARRIVE arrival guidelines and in strict adherence to the ethical principles of laboratory animals. Both male C57BL/6J (aged 8–10 weeks) and PD-L1 knockout mice (aged 8–10 weeks) were obtained from GemPharmatech™. All animals were maintained in a specific pathogen-free (SPF) animal room, under controlled conditions comprising a temperature of 23 ℃, humidity of 60%, 12-h light/12-h dark regime, with free access to food and drinking water. To construct a sepsis-induced ALI model, mice were first anesthetized with ketamine (75 mg/kg) and methyclothiazide (15 mg/kg), and a septic model mouse established by cecal ligation and perforation (one side entered the cecum and the other side passed through the cecal wall). CLP mice were anesthetized by intraperitoneal injection of 0.1 ml/10 g body weight of 4% chloral hydrate. A sterile environment was maintained during the procedure. A 1.5-cm incision was made in the lower abdominal region to expose the cecum. Using a 3–0 silk suture, the distal portion of the cecum was fully ligated 1 cm from the end. A single puncture was made with an 18-gauge needle before returning the cecum to the peritoneal cavity. The peritoneal wall and skin were closed using double sutures. Following surgery, the mice received a subcutaneous injection of 1 ml sterile saline (0.9%). Sham mice underwent the same abdominal incision and cecal exposure without ligation and puncture. Mice were provided with unlimited access to food and water post-procedure. After the sepsis model was established, indicated constructs were injected locally into the lungs via intranasal infusion, with 8 mice in each group. Mice were euthanized 24 h after completion of the treatment, and then peripheral blood and bronchoalveolar lavage fluid (BALF) as well as lung tissues were collected aseptically.

#### Hematoxylin and Eosin (H&E) staining

Fresh tissue was fixed in 4% paraformaldehyde for over 24 h and the target site leveled using a scalpel. The tissue was then dehydrated with an alcohol gradient and embedded in Type Histoplast PE generic paraffin wax (ThermoFisher). The wax block was sectioned to 4-μm thickness using a microtome. The sections were dewaxed twice in xylene for 5–10 min, transferred to anhydrous ethanol, followed by 90% ethanol, 70% ethanol, and finally distilled water. The sections were stained with hematoxylin (Raw Biological, E607317) for 3 min, rinsed with water, and then incubated with 0.25% hydrochloric acid alcohol. The sections were washed with distilled water, dipped in ammonia for 6 s, washed with running water for 5 min, then incubated for 20 min with eosin staining solution (Raw Biological, E607321). Next, the sections were dehydrated in 95% ethanol for 2 min, followed by three incubations in anhydrous ethanol (5 min for each incubation), then twice with xylene (5 min for each step). Finally, the sections were sealed with neutral gum or another sealing agent. An independent pathologist blindly assessed lung injury score using a 5-point scale, considering five criteria: perivascular and peribranchial inflammation, hyaline membranes, alveolar infiltrates, interstitial infiltrates, and alveolar hemorrhage. The scoring criteria for lung injury were as follows: 0 represented normal tissue, 1 indicated minimal inflammatory change, 2 denoted no evident damage to lung architecture, 3 represented thickening of alveolar septa, 4 indicated the formation of nodules or areas of pneumonitis causing distortion in the normal architecture, and 5 represented complete obliteration of the field.

#### ELISA

The proteins were detected using ELISA kits, according to the manufacturer’s instructions. In brief, cell medium, lung tissue, or BALF were collected and particulates removed by centrifugation. Samples were diluted and added to the wells to generate a colorimetric readout of the target protein concentration that was subsequently read as the OD at 450 nm using a microtiter plate reader. Concentration was calculated using the standard curve generated by the provided standards.

#### Statistical analysis

All statistical analyses were performed using SPSS version 21.0 and GraphPad Prism 5.0 software. Classification variables are expressed as counts or percentages and compared using χ 2 test. Continuous variables were presented as means ± standard deviation (SD), and compared between and among groups using Student’s *t*-test and one-way analysis of variance (ANOVA), respectively.

## Results

### Patients with sepsis and ALI exhibit differential expression of circAGFG1 and miR-195-5p relative to healthy controls

**Fig. 1:**
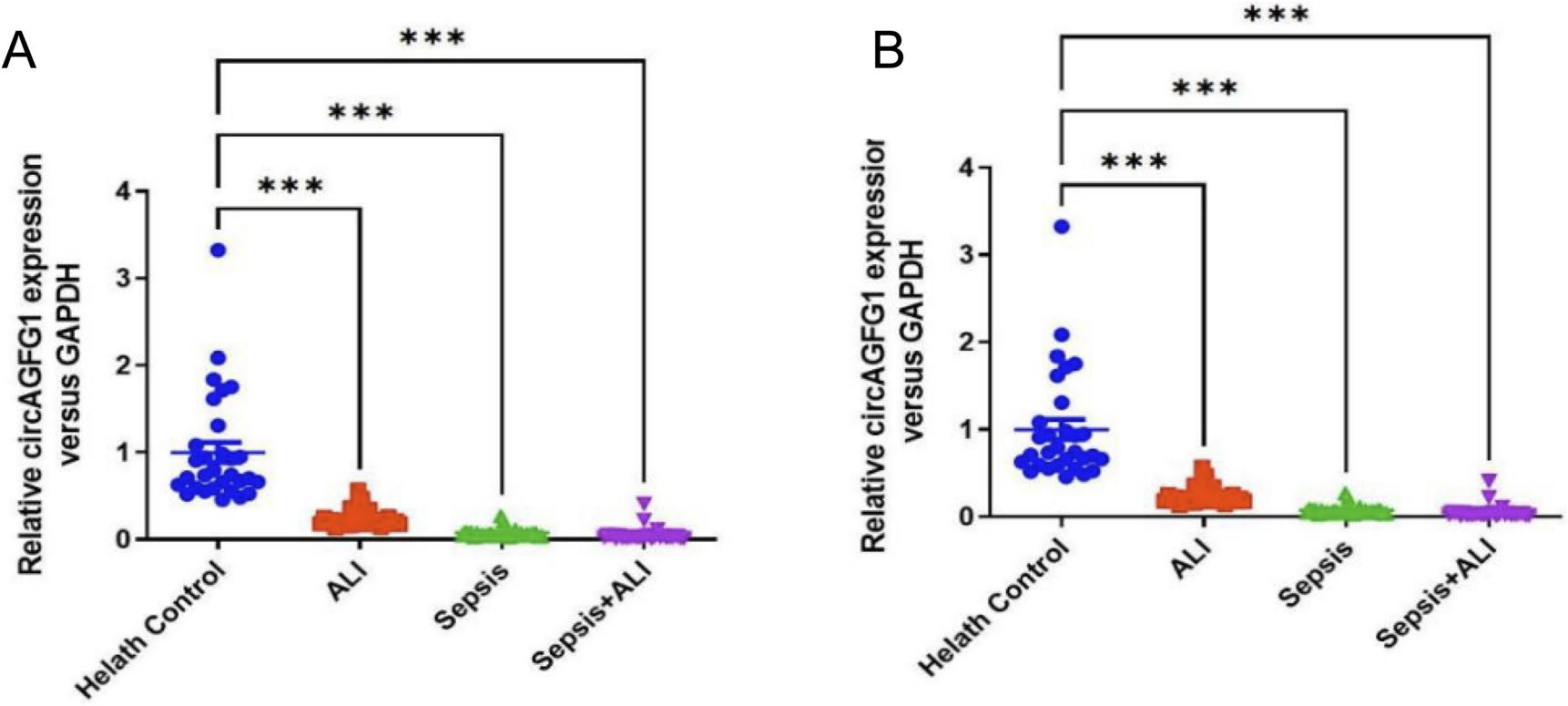
**A** circAGFG and **B** miR-195-5p expression levels in peripheral blood samples from patients with acute lung injury (ALI) and/or sepsis, determined by RT-PCR. circAGFG1 expression was normalized to GAPDH expression and miR-195-5p expression was normalized to U6 expression. *N* = 3, *p < 0.05, **p < 0.01, ***p < 0.001

To assess whether circAGFG1 and miR-195-5p play a role in sepsis and ALI, we used qRT-PCR to quantify expression of circAGFG1 and miR-195-5p mRNAs in peripheral blood of patients with ALI, and sepsis, as well as those with sepsis with ALI, relative to healthy controls. Results revealed significantly lower circAGFG1 expression in patients with ALI, sepsis, and sepsis with ALI than healthy controls (Fig. 1A), while miR-195-5p was significantly upregulated in all patient groups compared to healthy controls (Fig. 1B). These data suggest that circAGFG1 and miR-195-5p may be playing a role in sepsis response.

### circAGFG1 and PD-L1 expression affects miR-195-5p expression in epithelial lung cells

We first used a dual-luciferase assay to determine the interaction between circAGFG1, miR-195-5p, with PD-L1 mRNA in HEK293T cells. As expected, the wild-type miR-195-5p construct interacted with both the PD-L1 3’ untranslated region (UTR) and circAGFG1, while the non-coding (NC) miRNA construct and the mutated miR-195-5p did not interact with either PD-L1 or circAGFG1 (Supplemental Fig. S1). Next, we generated over-expression (OE) and silencing RNA (siRNA) constructs targeting circAGFG1 and PD-L1, then prepared three siRNA constructs for each target and used the most effective construct in subsequent experiments (Supplemental Fig. S2).

**Fig. 2:**
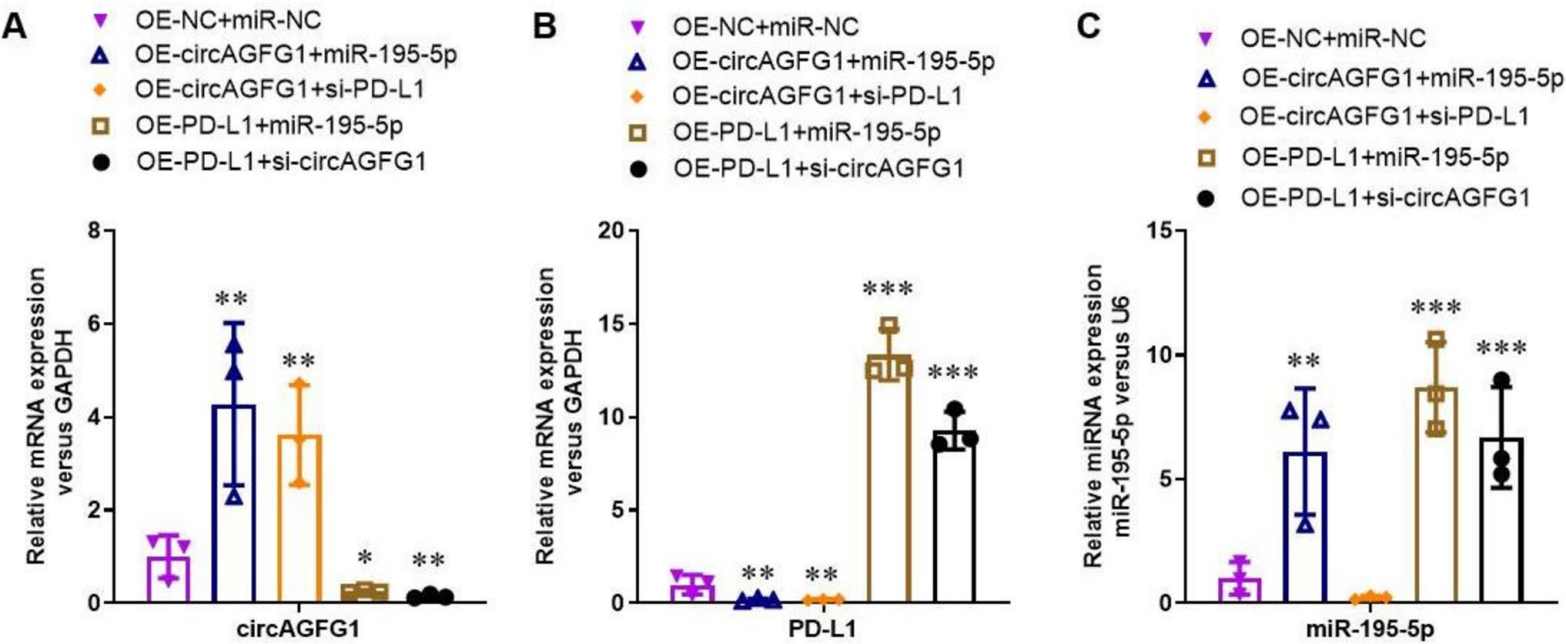
Relative expression of **A** circAGFG1, **B** PD-L1, and **C** miR-195-5p RNA in CalU-3 cells as determined by RT-PCR. PD-L1 and circAGFG1 expression levels were normalized to GAPDH expression and miR-195-5p expression was normalized to U6 expression. *N* = 3, *p < 0.05, **p < 0.01, ***p < 0.001

Next, we investigated the function of the circAGFG1/miR-195-5p/PD-L1 axis in epithelial lung cells by simulating sepsis conditions in Calu-3 cells with LPS treatment. Specifically, cells were transfected with OE constructs for circAGFG1, PD-L1, and NC control; miR-195-5p or miR-NC constructs; and siRNA constructs for PD-L1 and circAGFG1. qRT-PCR targeting circAGFG1, PD-L1, and miR-195-5p, revealed that transfection OE-circAGFG1 significantly upregulated circAGFG1 expression, while si-circAGFG1 significantly downregulated its expression (Fig. 2A). Notably, the expression of miR-195-5p in addition to OE-circAGFG1 did not affect the expression of circAGFG1 mRNA. Similarly, OE-PD-L1 significantly upregulated the expression of PD-L1 mRNA (Fig. 2B) or protein (Supplemental Fig. S3) compared to NC constructs, regardless of co-transfection with si-circAGFG1 or miR-195-5p. As expected, miR-195-5p was significantly upregulated when cells were transfected with a construct harboring miR-195-5p. However, high miR-195-5p expression was also recorded in cells co-transfected with OE-PD-L1 and si-circAGFG1 (Fig. 2C). Collectively, these results indicated that endogenous levels of circAGFG1 may suppress miR-195-5p expression, or that higher PD-L1 expression may lead to negative feedback by upregulating miR-195-5p expression.

### The circAGFG1/miR-195-5p/PD-L1 axis regulates short-term cell viability and apoptosis

**Fig. 3:**
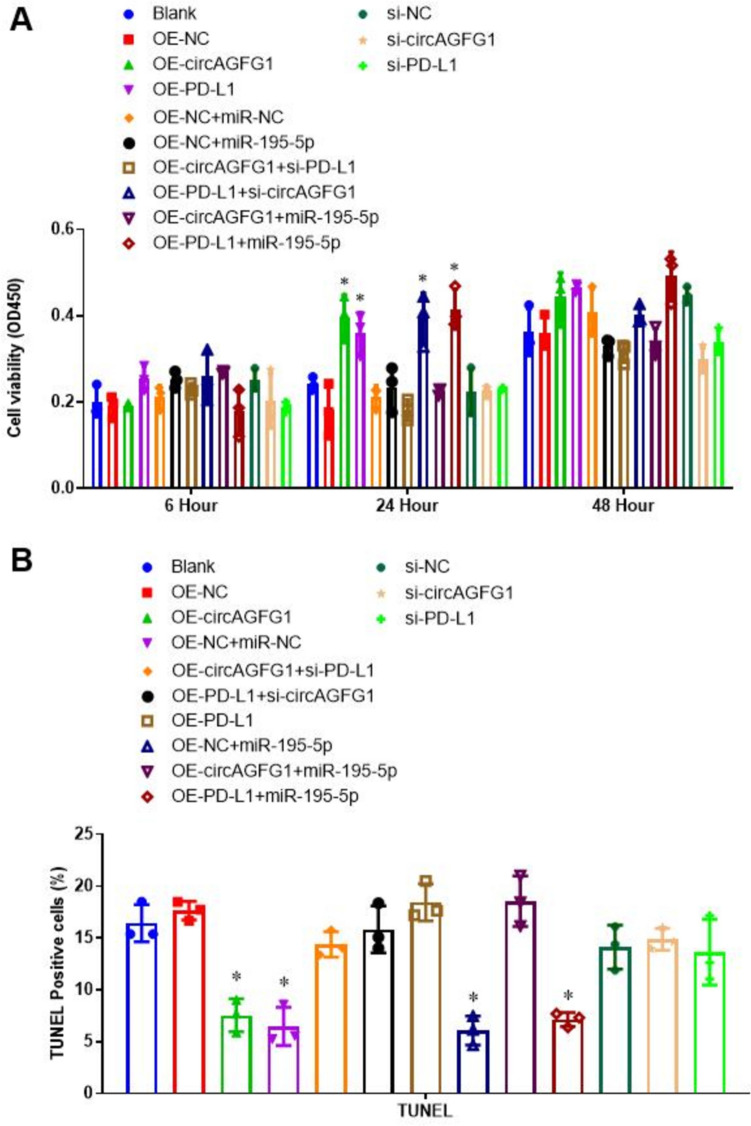
**A** Cell viability and **B** apoptosis (TUNEL staining) in Calu-3 cells transfected with the indicated construct(s). *N* = 3, *p < 0.05, **p < 0.01, ***p < 0.001

We employed CCK-8 and TUNEL staining assays to assess cell viability and apoptosis, respectively. Results revealed significant differences in cell viability at 24 h, with PD-L1 over-expression promoting cell viability regardless of circAGFG1 and miR-195-5p levels (Fig. 3A). Similarly, over-expression of circAGFG1 promoted cell viability, although it blocked circAGFG1-induced increase when combined with si-PD-L1 or miR-195-5p. This implied that the circAGFG1-mediated effect on cell viability involves the downregulation of miR-195-5p expression and enhanced PD-L1 regulation, consistent with our model results. However, we found no statistically significant differences in cell viability at 48 h, indicating that the circAGFG1/miR-195-5p/PD-L1 axis may only regulate short-term cell viability. Alternatively, cells may begin to silence target transfected constructs, thereby confounding the effects of the expressed constructs at longer time points. Results of the TUNEL staining assay performed 24 h after LPS treatment, revealed 24-h cell viability, with decreased apoptosis in cells with increased expression of PD-L1, either through OE-PD-L1 or OE-circAGFG1 expression (Fig. 3B and Supplemental Fig. S4).

### High PD-L1 expression mediates the upregulation of occludin and E-cadherin proteins

**Fig. 4:**
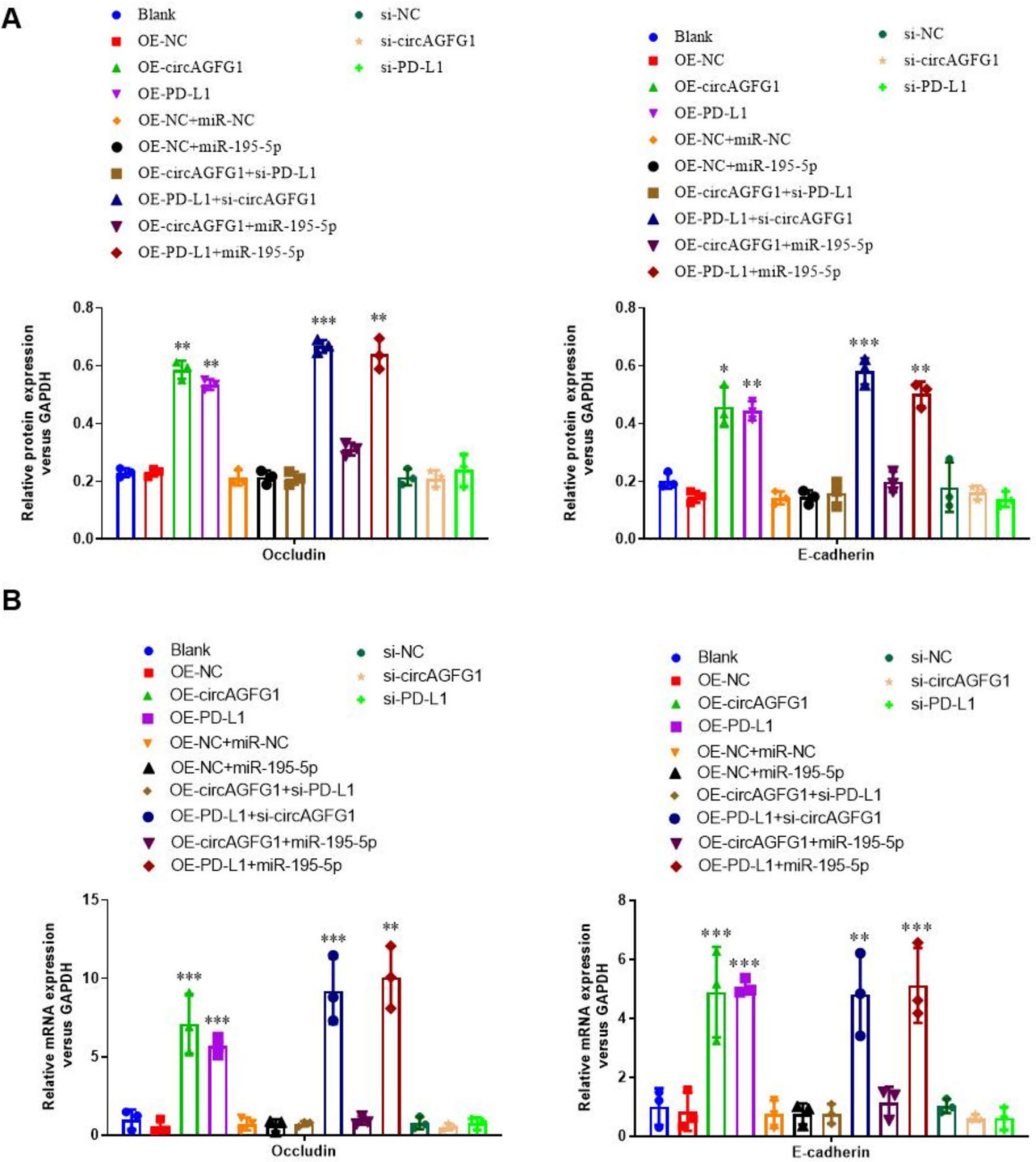
Occludin and E-cadherin expression relative to GAPDH in Calu-3 cells transfected with the indicated construct(s) by **A** Western blot and **B** RT-PCR. *N* = 3, *p < 0.05, **p < 0.01, ***p < 0.001

Previous studies have demonstrated that cellular adhesion proteins, occludin and E-cadherin are downregulated by inflammatory signals [50–52]. Particularly, occludin downregulation is associated with epithelial cell survival in the context of inflammation. In the present study, we analyzed the expression of occludin and E-cadherin proteins (Fig. 4A and Supplemental Fig. S5) and mRNA (Fig. 4B) in Calu-3 cells treated with LPS. Results showed that both protein and mRNA of both factors were upregulated with an increase in PD-L1 expression, including PD-L1 expression induced by OE-circAGFG1. As expected, the addition of miR-195-5p blocked OE-circAGFG1 effects. Interestingly, si-PD-L1 had no effect on neither occludin nor E-cadherin expression, indicating that other pathways may also be playing a role in maintaining base levels of occludin and E-cadherin under sepsis-like conditions.

### Upregulation of PD-L1 in Calu-3 cells downregulated inflammatory cytokine expression and suppressed the viability of Th17 cells in a co-culture system

**Fig. 5:**
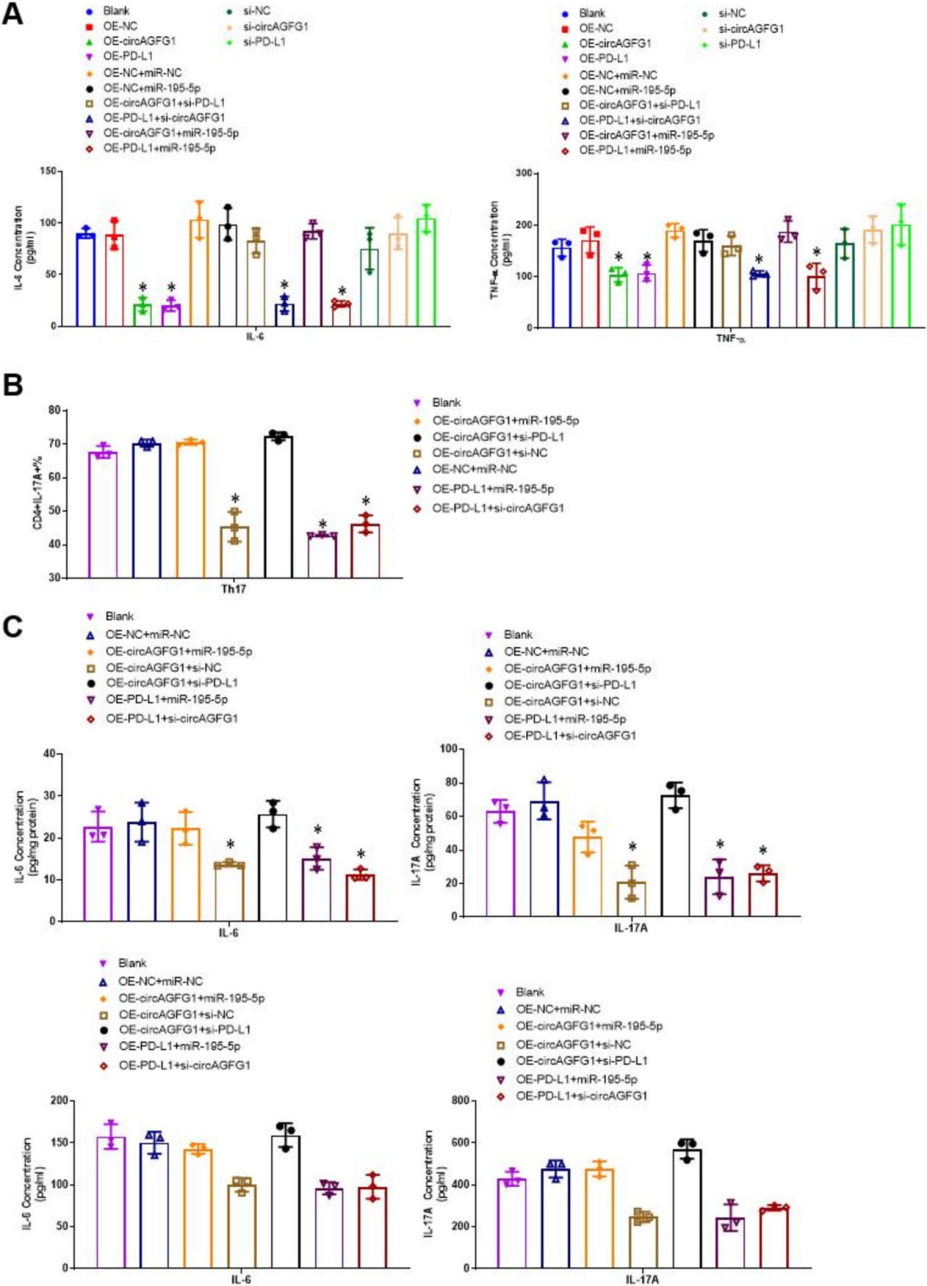
Cytokine expression and Th17 differentiation in Calu-3 and induced Th17 cells. **A** IL-6 and TNF-α concentrations in cell medium of Calu-3 cells expressing the indicated construct(s) and treated with LPS. **B** Th17 differentiation in T cells co-cultured with Calu-3 cells expressing the indicated construct(s) and treated with LPS. C) IL-6 and IL-17A concentrations in cell lysate (top) or supernatant (bottom) from Th17 cells co-cultured with Calu-3 cells expressing the indicated construct(s). *N* = 3, *p < 0.05, **p < 0.01, ***p < 0.001

To investigate the role of the circAGFG1/miR-195-5p/PD-L1 axis in the interaction between lung epithelial cells and immune cells, we first used ELISA to measure cytokine concentrations in media with transfected Calu-3 cells. Results showed that over-expressing PD-L1 suppressed IL-6 and TNF-α concentration in the medium (Fig. 5A). Similarly, over-expression of circAGFG1 also decreased inflammatory cytokine concentrations, although this was reversed following co-transfection with miR-195-5p. Next, we induced Th17 cells from naïve human CD4+ T cells (Supplemental Fig. S6), then co-cultured them with transfected Calu-3 cells and measured the proportion of cells expressing IL-17 with FACS. Results showed that co-culturing with Calu-3 cells over-expressing PD-L1 suppressed the number of IL-17 + cells compared to controls (Fig. 5B and Supplemental Fig. S7). On the other hand, over-expression of circAGFG1 significantly mediated a decrease in the number of IL-17 + cells. Notably, siRNA-mediated PD-L1 and co-transfection with miR-195-5p effectively reversed the effect of the over-expression of circAGFG1. Analysis of IL-6 and IL-17 concentrations in cell medium revealed a similar pattern, significantly decreasing with increased PD-L1 expression (Fig. 5C).

**Fig. 6:**
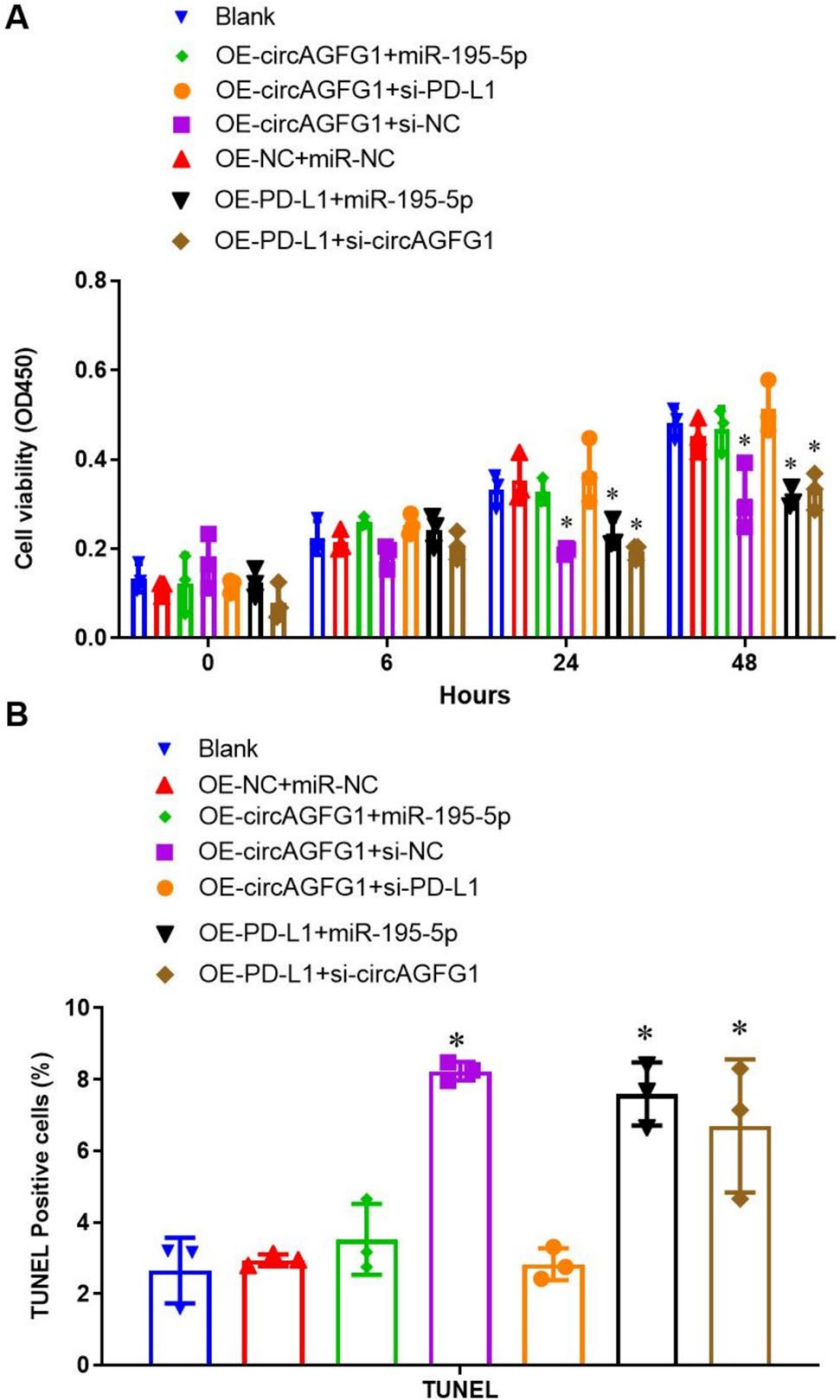
**A** Cell viability and **B** apoptosis (TUNEL staining) of Th17 cells co-cultured with Calu-3 cells expressing the indicated constructs. *N* = 3, *p < 0.05, **p < 0.01, ***p < 0.001

Next, we evaluated the effect of co-culture on viability and apoptosis of Th17 cells. Results showed that Th17 cells co-cultured with Calu-3 cells over-expressing PD-L1 had significantly lower cell viability at 24 and 48 h (Fig. 6A), and significantly higher TUNEL-positive cells, indicative of elevated apoptosis (Fig. 6B). Similarly, Th17 cells co-cultured with Calu-3 cells over-expressing circAGFG1 exhibited significantly lower cell viability and higher numbers of apoptotic cells. However, co-transfection with miR-195-5p or siPD-L1 reversed the high circAGFG1 expression.

### PD-L1 expression is necessary for circAGFG1 and miR-195-5p regulation of inflammatory cytokines in a sepsis-induced ALI mouse model

**Fig. 7:**
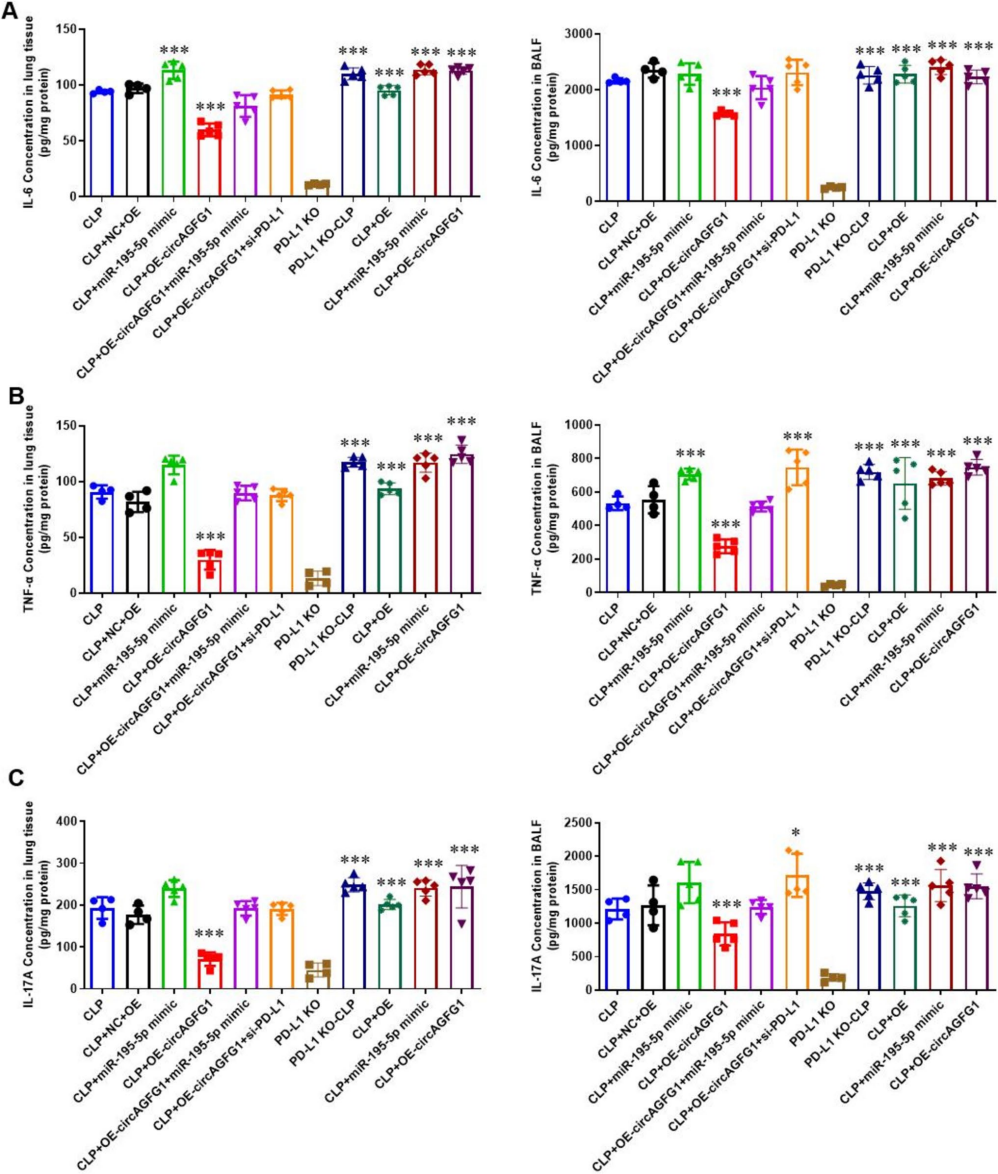
Cytokine (**A** IL-6, **B** TNF-alpha, **C** IL-17) in lung tissue from wild-type (right) or PD-L1 knockout (KO, left) mice with cecal ligation and puncture (CLP)-induced sepsis. *N* = 3, *p < 0.05, **p < 0.01, ***p < 0.001

**Fig. 8:**
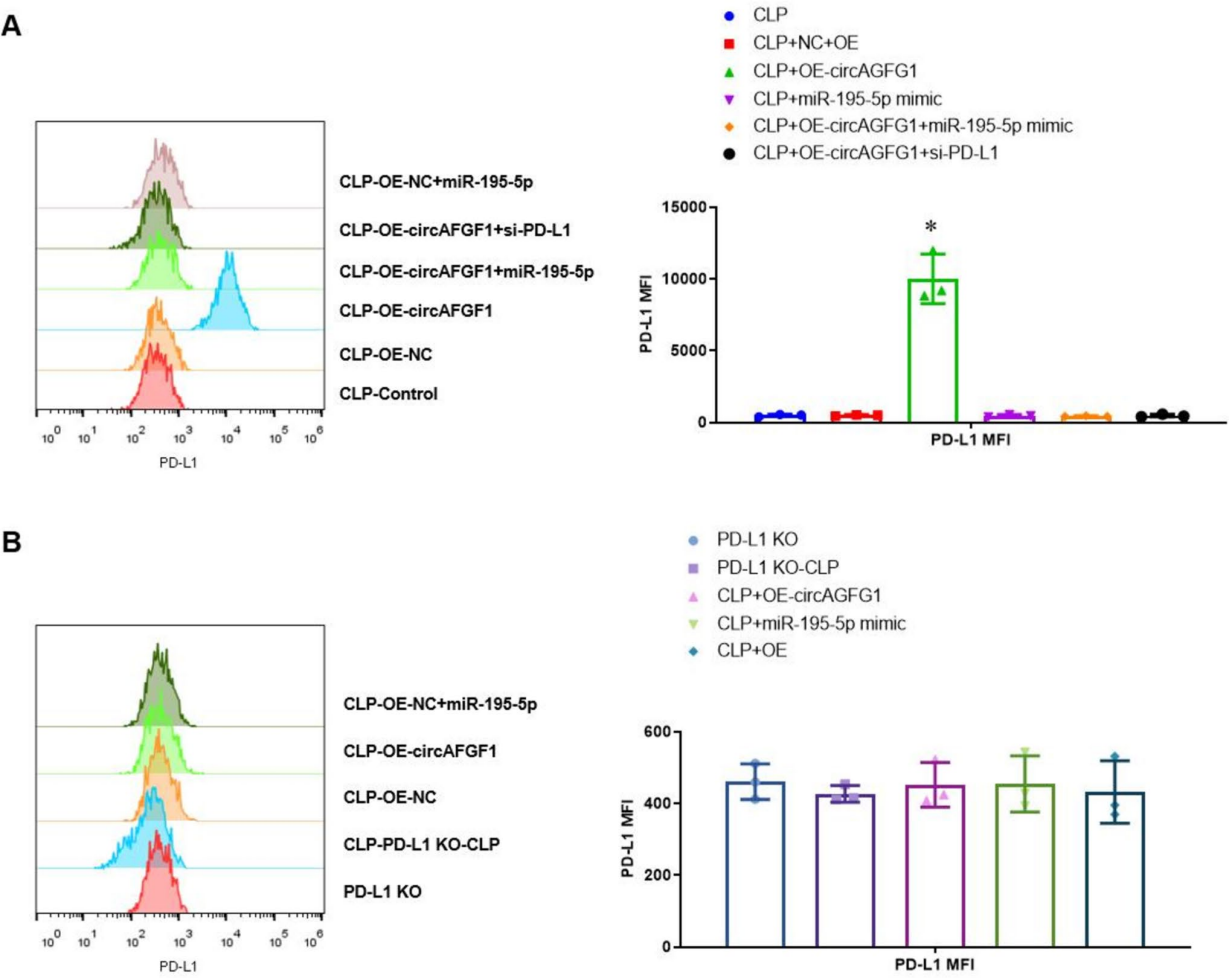
Flow cytometry showing PD-L1 expression in lung tissue from A) wild-type or B) PD-L1 knockout (KO) mice with cecal ligation and puncture (CLP)-induced sepsis. *N* = 3, *p < 0.05, **p < 0.01, ***p < 0.001

To validate the effects observed in cell lines in vivo, we established a cecal ligation and puncture (CLP) mouse model of sepsis and ALI in male C57BL/6 J and PD-L1 knockout mice (PD-L1 KO) treated locally with the indicated constructs. Results of H&E staining of mouse lung tissues showed that treatment with the constructs and CLP affected tissue organization (Supplemental Fig. S8A and B). Next, we employed the PD-L1 interference construct to test efficacy of different doses for local injection (Supplemental Fig. S8C), then measured concentrations of various inflammatory cytokines, namely IL-6, TNF-α, and IL-17, in lung tissue and BALF in wild-type and PD-L1 KO mice. As expected, CLP significantly upregulated inflammatory cytokine levels in both tissue and BALF in PD-L1 KO mice (Fig. 7A–C, left), while expression of either circAGFG1 or miR-195-5p had no effect on cytokine levels in the absence of PD-L1. Notably, over-expression of circAGFG1 in mice expressing PD-L1 significantly suppressed concentrations of all three cytokines in both tissue and BALF, while co-expression of miR-195-5p or siPD-L1 reversed the effect of circAGFG1, as evidenced in cultured lung epithelial and Th17 cells (Fig. 7A–C, right). Flow cytometry results indicated that over-expression of circAGFG1 significantly upregulated PD-L1 expression in mouse lung epithelial cells (Fig. 8A and Supplemental Fig. S9). With or without over-expression of circAGFG1 treatment, the PD-L1 levels in wild-type lung epithelial cells treated with CLP had comparable levels of PD-L1 expression with those in PD-L1 KO mice (Fig. 8B and Supplemental Fig. 10), indicating that sepsis may suppress PD-L1 expression in lung epithelial cells through miR-195-5p, a phenomenon that may be counteracted by treatment with exogenous circAGFG1.

### circAGFG1-modulated PD-L1 expression suppresses Th17 differentiation in CLP-treated mouse lung tissue

**Fig. 9:**
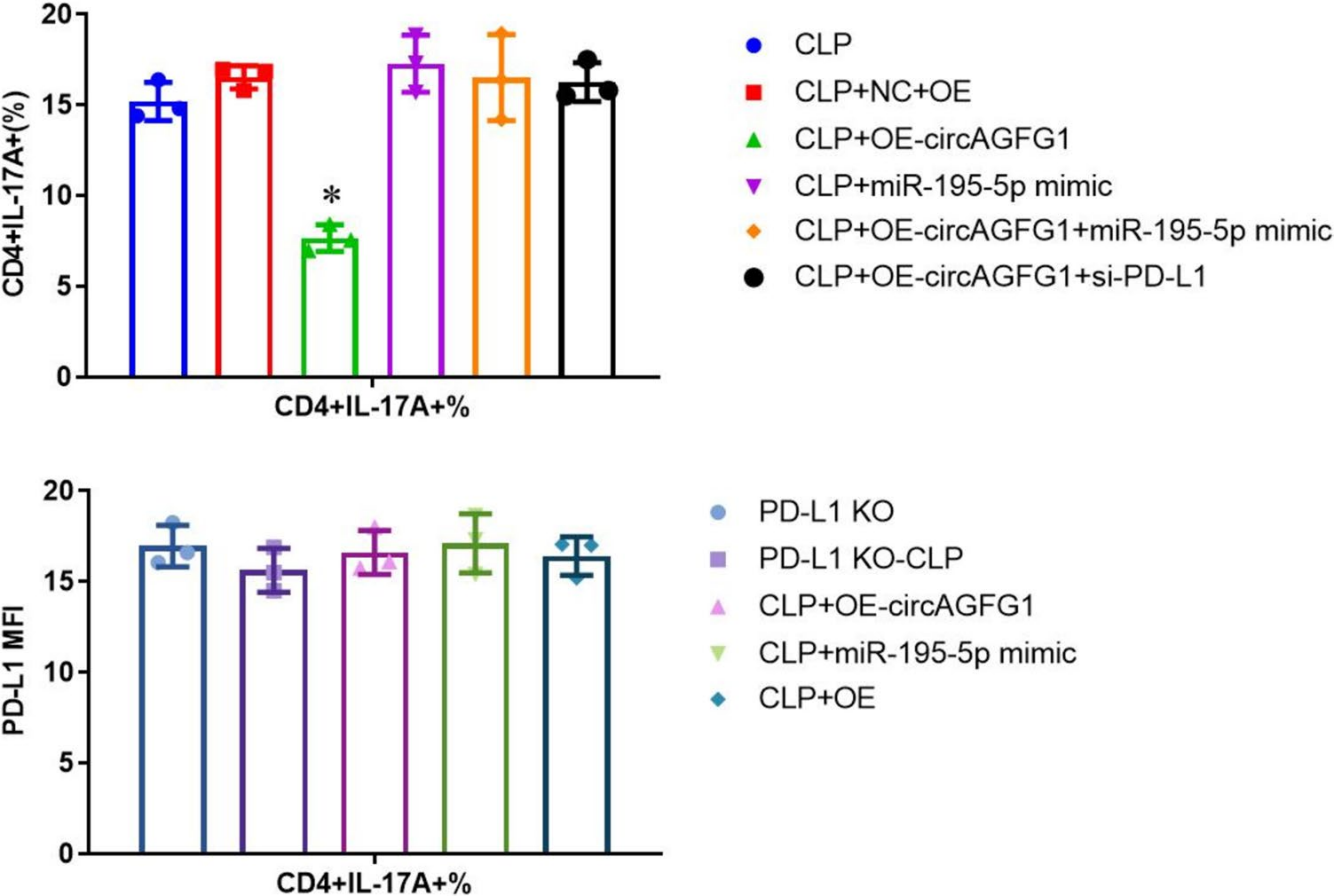
Quantification of flow cytometry results for CD4+ T cell IL-17 levels in lung tissue from wild-type (top) or PD-L1 knockout (KO, bottom) mice with cecal ligation and puncture (CLP)-induced sepsis. (Full FACS plots in supplement.) *N* = 3, *p < 0.05, **p < 0.01, ***p < 0.001

High PD-L1 expression and low cytokine concentrations in lung tissue over-expressing circAGFG1 correlated with low proportions of IL-17 + T cells in the lungs. This indicates that PD-L1 expression is necessary for the over-expression of circAGFG1 on Th17 cell differentiation. We observed no effect in PD-L1 KO mice, while co-expression of exogenous miR-195-5p or siPD-L1 prevented the effect of circAGFG1 over-expression (Fig. 9 and Supplemental Fig. S11).

### Expression of occludin and E-cadherin proteins increase with upregulation of PD-L1 in CLP-treated mouse lung tissue

**Fig. 10:**
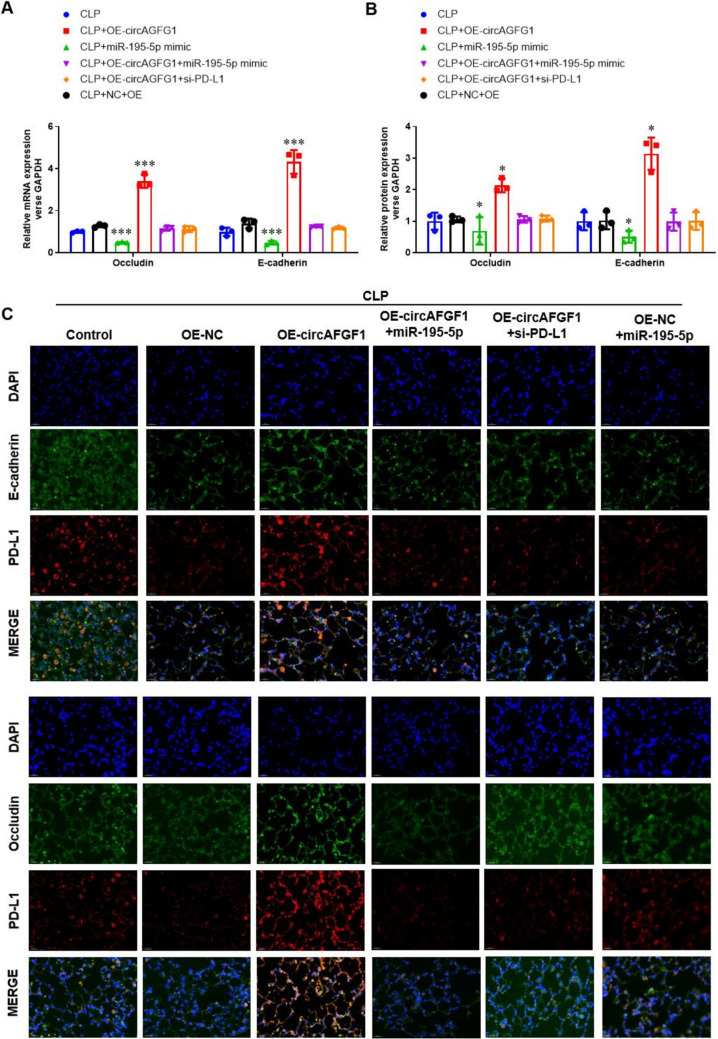
Occludin and E-cadherin expression in lung tissue from wild-type mice with cecal ligation and puncture (CLP)-induced sepsis. **A** RT-PCR quantification of occludin and E-cadherin expression level relative to GAPDH, **B** Western blot quantification of occludin and E-cadherin expression relative to GAPDH, and **C** immunofluorescence images of E-cadherin, occludin, and PD-L1 expression. *N* = 3, *p < 0.05, **p < 0.01, ***p < 0.001

**Fig. 11:**
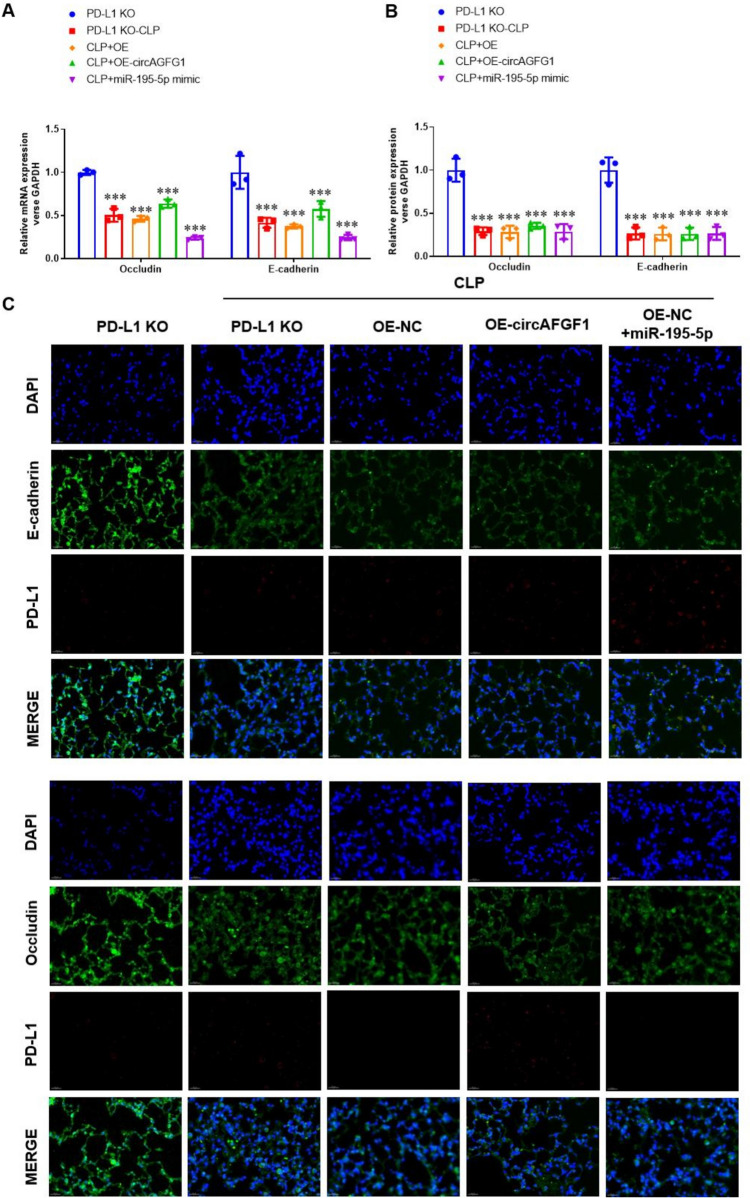
Occludin and E-cadherin expression in lung tissue from PD-L1 knockout (KO) mice with cecal ligation and puncture (CLP)-induced sepsis. **A** RT-PCR quantification of occludin and E-cadherin expression relative to GAPDH, **B** Western blot quantification of occludin and E-cadherin expression relative to GAPDH, and **C** immunofluorescence images of E-cadherin, occludin, and PD-L1 expression. *N* = 3, *p < 0.05, **p < 0.01, ***p < 0.001

Next, we evaluated the effects of the circAGFG1/miR-195-5p/PD-L1 axis on occludin and E-cadherin expression in CLP-treated wild-type and PD-L1 KO mouse lung tissues. Results revealed that over-expression of circAGFG1 upregulated expression of occludin and E-cadherin mRNA in wild-type mice (Fig. 10A) and protein expression (Fig. 10B, C and Supplemental Fig. S12A). Exogenous miR-195-5p and siPD-L1 not only downregulated occludin and E-cadherin expression, but also blocked the effect of circAGFG1 over-expression. On the other hand, CLP treatment downregulated the expression of occludin and E-cadherin mRNA in PD-L1 KO mice (Fig. 11A) and protein expression (Fig. 11B, C and Supplemental Fig. S12B), indicating that low occludin and E-cadherin expression is part of the endogenous sepsis response, even in the absence of PD-L1. As expected, over-expression of circAGFG1 and exogenous miR-195-5p conferred no significant effects on occludin and E-cadherin expression in the absence of PD-L1.

## Discussion

Sepsis, characterized by a dysregulated host response to infection, is a leading cause of morbidity and mortality in critically ill patients. One of the most severe complications of sepsis is ALI, which can progress to ARDS. ALI and ARDS are associated with high mortality rates due to the resultant respiratory failure and multiple organ dysfunctions. Despite advancements in critical care, the therapeutic options for sepsis-induced ALI remain limited and largely supportive, highlighting the urgent need for novel therapeutic strategies [15, 16, 53].

Recent advances in molecular biology have unveiled the crucial roles of non-coding RNAs, such as circRNAs and miRNAs, in the regulation of gene expression and cellular processes. CircRNAs, due to their stable circular structure, can act as molecular sponges, sequestering miRNAs and preventing them from interacting with their target messenger RNAs (mRNAs). This study focuses on the circAGFG1/miR-195-5p/PD-L1 axis, exploring its potential role in the pathogenesis of sepsis-induced ALI [54].

Our findings indicate that circAGFG1 expression is significantly reduced, while miR-195-5p expression is markedly increased in the peripheral blood of patients with sepsis, ALI, and sepsis complicated with ALI, compared to healthy controls [22, 55]. This differential expression suggests a potential regulatory interplay between circAGFG1 and miR-195-5p. The downregulation of circAGFG1 may result in the release of miR-195-5p, which could then exert its regulatory effects on target genes, including PD-L1.

The dual-luciferase reporter assays conducted in this study demonstrated a direct interaction between miR-195-5p and the 3’ UTR of PD-L1 mRNA. In addition, miR-195-5p was found to bind to circAGFG1, suggesting that circAGFG1 functions as a molecular sponge for miR-195-5p. This sponging effect was further confirmed through over-expression and silencing experiments, which showed that circAGFG1 modulates PD-L1 expression by sequestering miR-195-5p [56, 57].

PD-L1 is an immune checkpoint protein that plays a critical role in modulating immune responses. By inhibiting the interaction between miR-195-5p and PD-L1 mRNA, circAGFG1 prevents the miRNA-mediated downregulation of PD-L1, thereby influencing immune responses in the lung epithelium [58–60]. This regulatory mechanism highlights the importance of the circAGFG1/miR-195-5p/PD-L1 axis in the context of sepsis-induced ALI.

Inflammatory cytokines and Th17 cells are pivotal in the immune response during sepsis and ALI. Th17 cells, a subset of CD4+ T cells, are known for their role in autoimmunity and inflammation. Our study revealed that the circAGFG1/miR-195-5p/PD-L1 axis significantly affects the expression of inflammatory cytokines and the differentiation of Th17 cells.

In co-culture experiments involving Th17 cells and transfected Calu-3 cells, we observed that modulation of circAGFG1 and miR-195-5p levels influenced the production of cytokines such as IL-17 and IL-22, which are key mediators of Th17 cell function [10]. These findings suggest that the circAGFG1/miR-195-5p/PD-L1 axis not only affects PD-L1 expression but also has broader implications for the inflammatory milieu in sepsis-induced ALI.

**Fig. 12:**
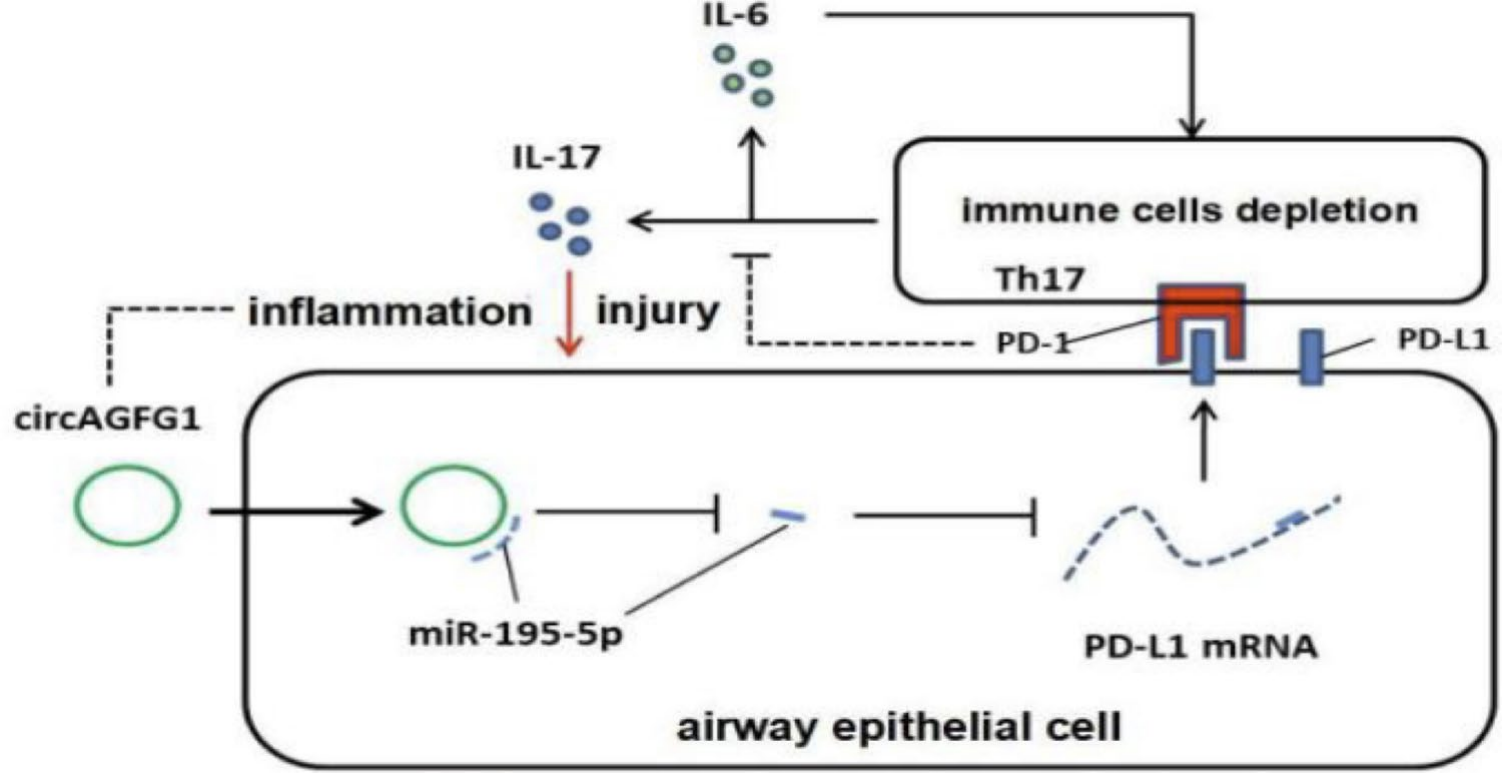
**A** Pathway diagram showing the proposed circAGFG1/miR-195-5p/PD-L1 axis

The regulatory role of the circAGFG1/miR-195-5p/PD-L1 axis in inflammation and epithelial cell survival presents a promising therapeutic target for sepsis-induced ALI. Modulating this axis could potentially mitigate the excessive inflammatory response and improve lung epithelial cell survival, thereby reducing lung injury and improving patient outcomes（Fig. 12）. Given the limited efficacy of current treatments for sepsis-induced ALI/ARDS, targeting this molecular pathway offers a novel therapeutic approach.

## Limitations and future directions

While our study provides valuable insights into the role of the circAGFG1/miR-195-5p/PD-L1 axis in sepsis-induced ALI, several limitations should be addressed in future research. First, our findings were based on in vitro and murine models, and further validation in human clinical samples is necessary to confirm the clinical relevance of these molecular interactions. In addition, the exact mechanisms by which circAGFG1 and miR-195-5p regulate PD-L1 expression and the downstream effects on immune responses require more detailed exploration. Understanding these mechanisms will be crucial for developing targeted therapies that can effectively modulate this axis in clinical settings. In addition, although we used the Calu-3 cell line as a model for lung epithelial cells, it is important to note that Calu-3 cells are derived from a non-small-cell lung carcinoma. While they retain some features of lung epithelium, they may not fully represent normal, non-cancerous epithelial cells. Future studies should include primary human epithelial cells for validation to better assess the physiological relevance of this axis in normal lung epithelium.

## Conclusion

Our study demonstrates that the circAGFG1/miR-195-5p/PD-L1 axis plays a significant role in regulating inflammatory responses and epithelial cell survival in sepsis-induced ALI. The differential expression of circAGFG1 and miR-195-5p in sepsis and ALI patients highlights their potential as biomarkers and therapeutic targets. Targeting this axis offers a promising strategy to mitigate inflammation and improve outcomes in sepsis-induced ALI. Further research is needed to validate these findings in clinical settings and to develop targeted therapies that can effectively modulate this regulatory pathway.

## Supplementary Information

Below is the link to the electronic supplementary material.

Supplementary file1 (DOCX 2582 kb)

## Data Availability

The data used for this research are available from the corresponding author on reasonable request and subject to Institutional Review Board guidelines.
