# Supplementary material for: Activation of the circAGFG1/miR-195-5p/PD-L1 axis induces lung injury in sepsis
Source: Hum Cell. 2025 Jul 14;38(5):129. doi: 10.1007/s13577-025-01258-z (PMC12259802; doi:10.1007/s13577-025-01258-z)
Supplement: Supplementary file 1 — Supplementary file1 (DOCX 2582 kb) [file 13577_2025_1258_MOESM1_ESM.docx]

**The mechanism of circAGFG1/miR-195-5p/PD-L1 in sepsis-induced acute lung injury**

**Supplementary Figures**


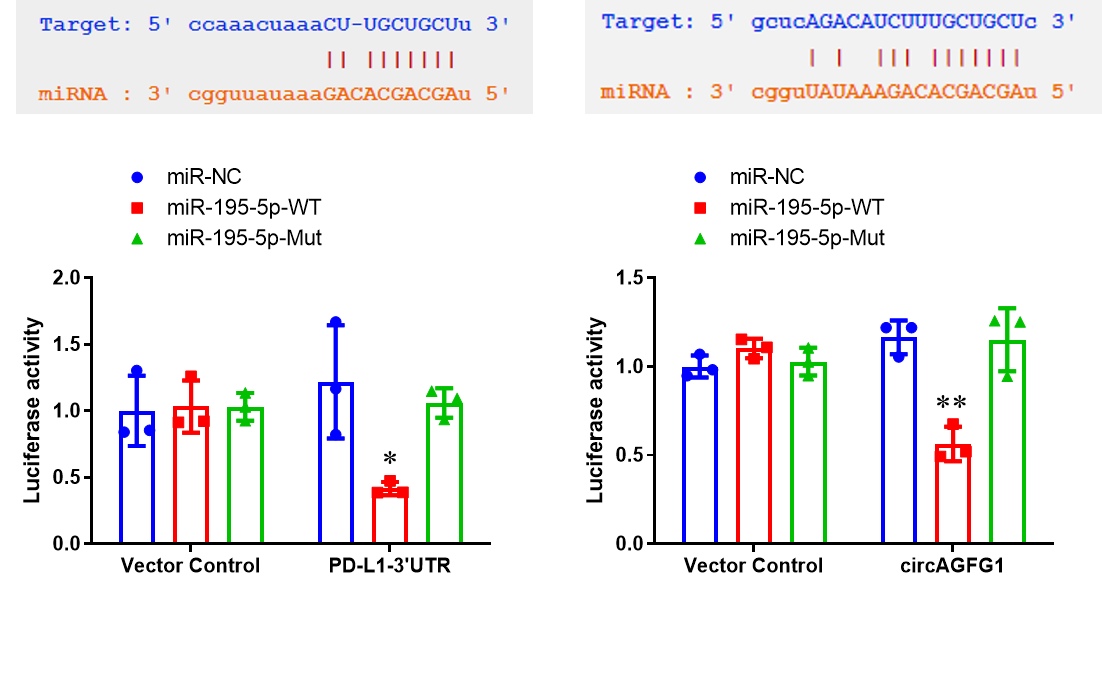


**Supplemental Figure 1.** Proposed interaction sequences and luciferase assays for interactions between miR-195-5p and the 3’ UTR of PD-L1 mRNA (left) or circAGFG1 (right). N=3.


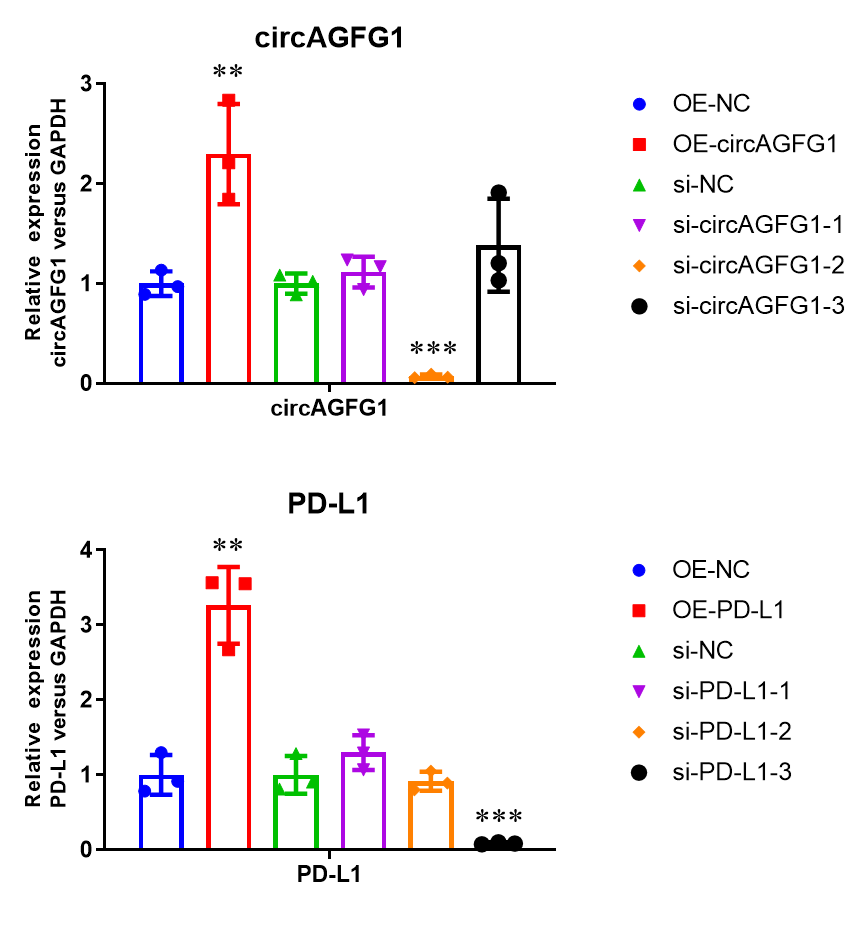


**Supplemental Figure 2.** Silencing RNA construct development for circAGFG1 and PD-L1. Expression determined by RT-PCR. N=3.

**
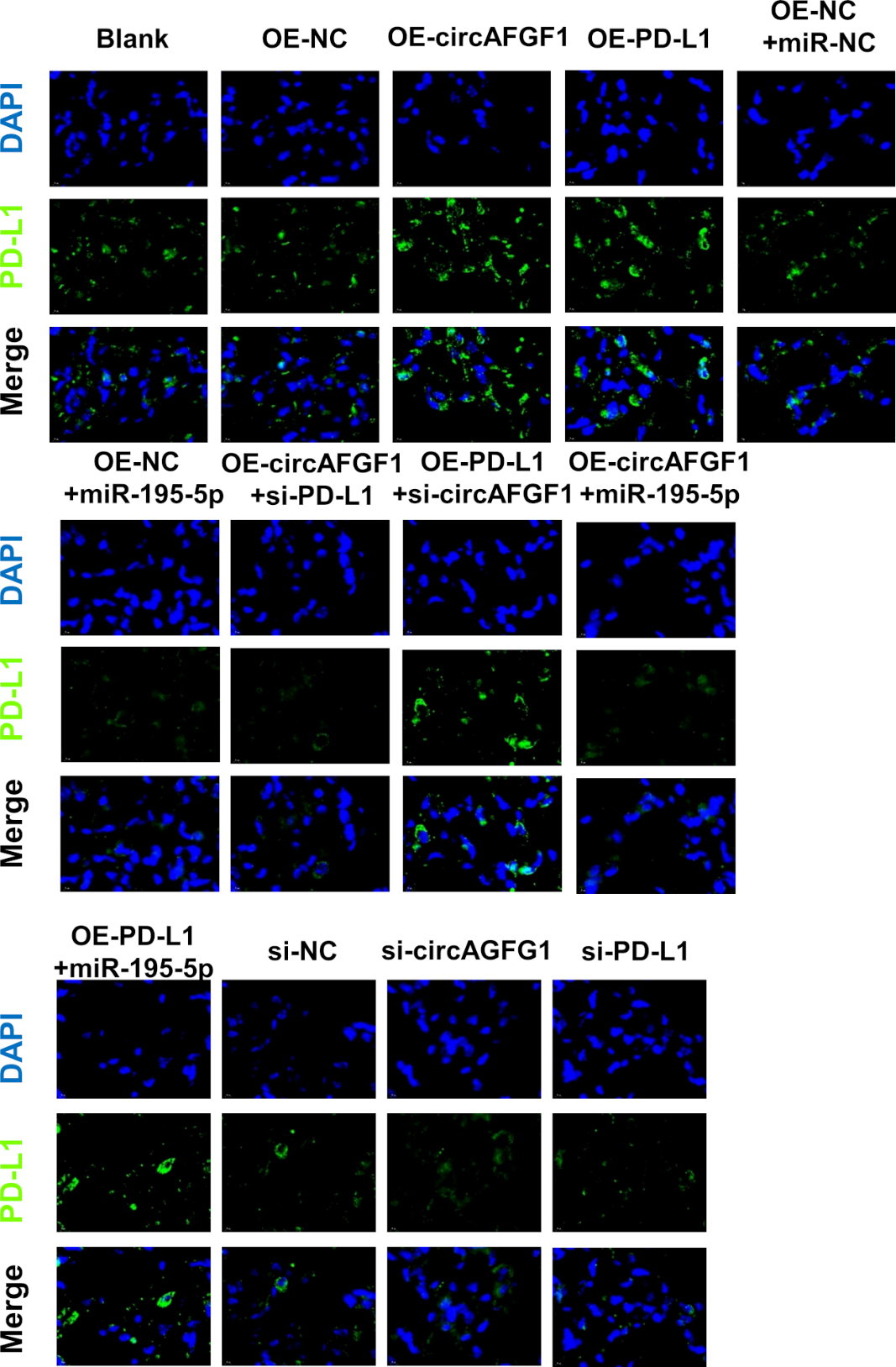
**

**Supplemental Figure 3.** Immunofluorescent images for PD-L1 expression in Calu-3 cells. N=3.

**
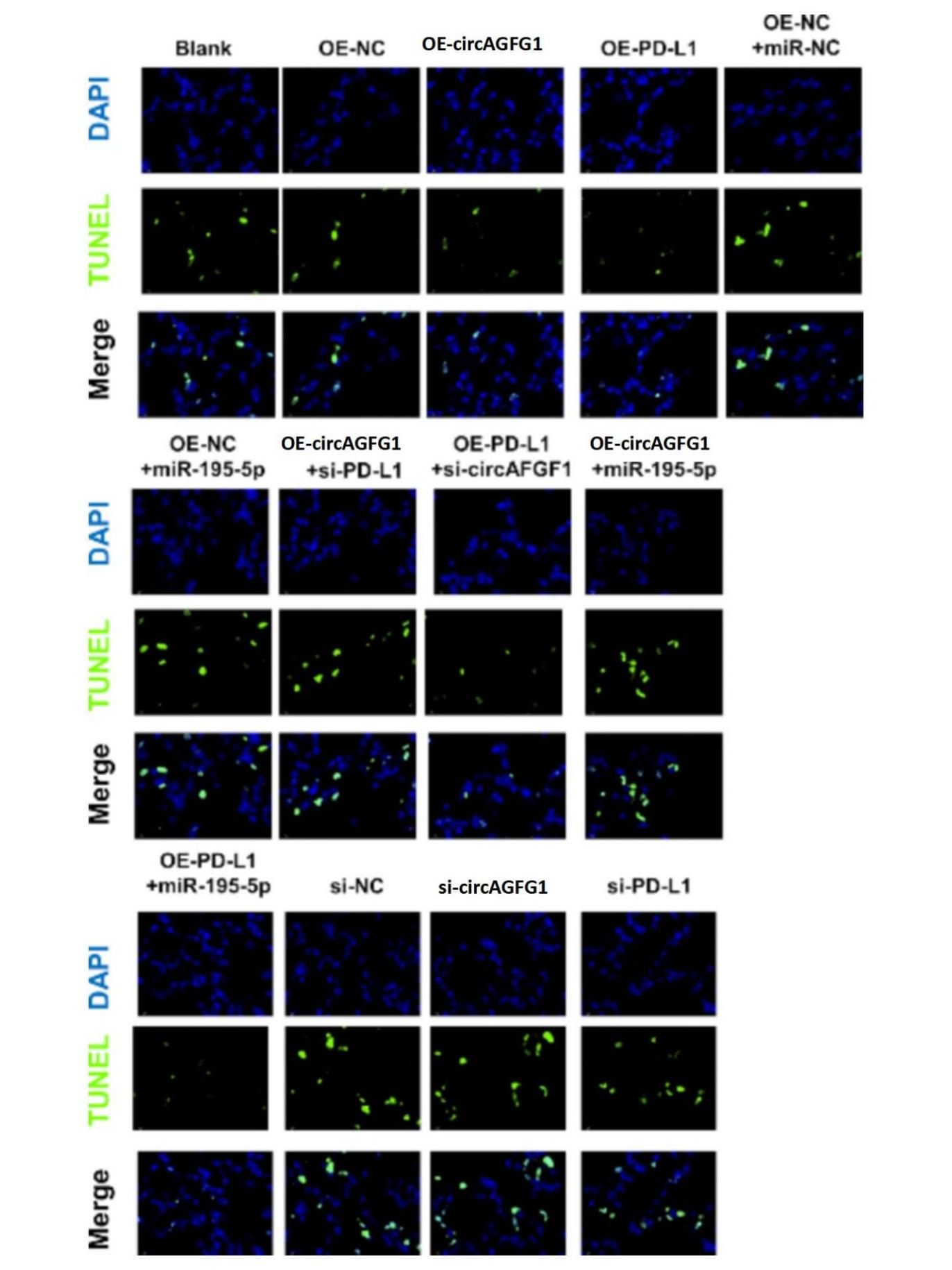
**

**Supplemental Figure 4.** TUNEL staining images for Calu-3 cells. N=3.

**
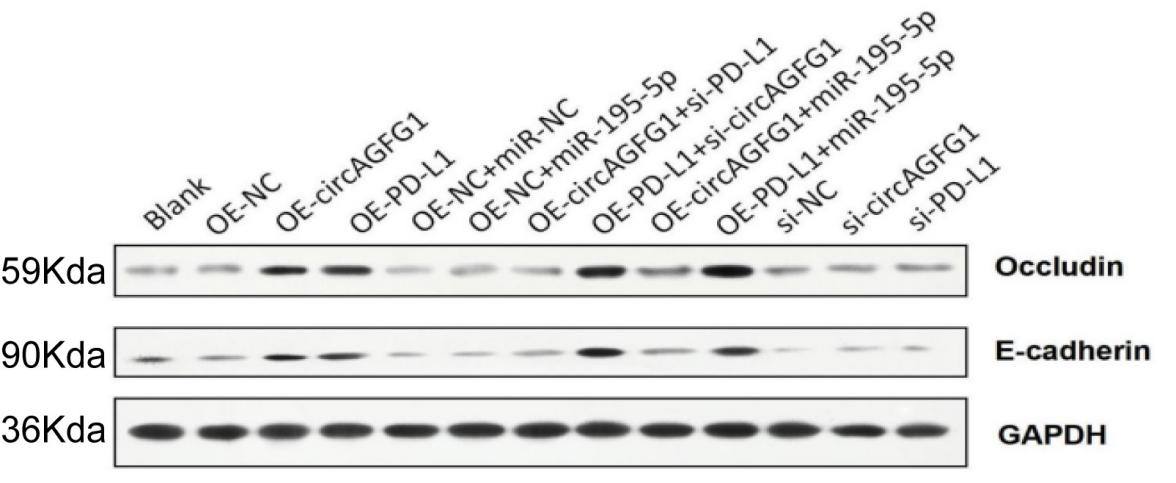
**

**Supplemental Figure 5.** Western blots for occludin, E-cadherin, and GAPDH expression in Calu-3 cells. N=3.


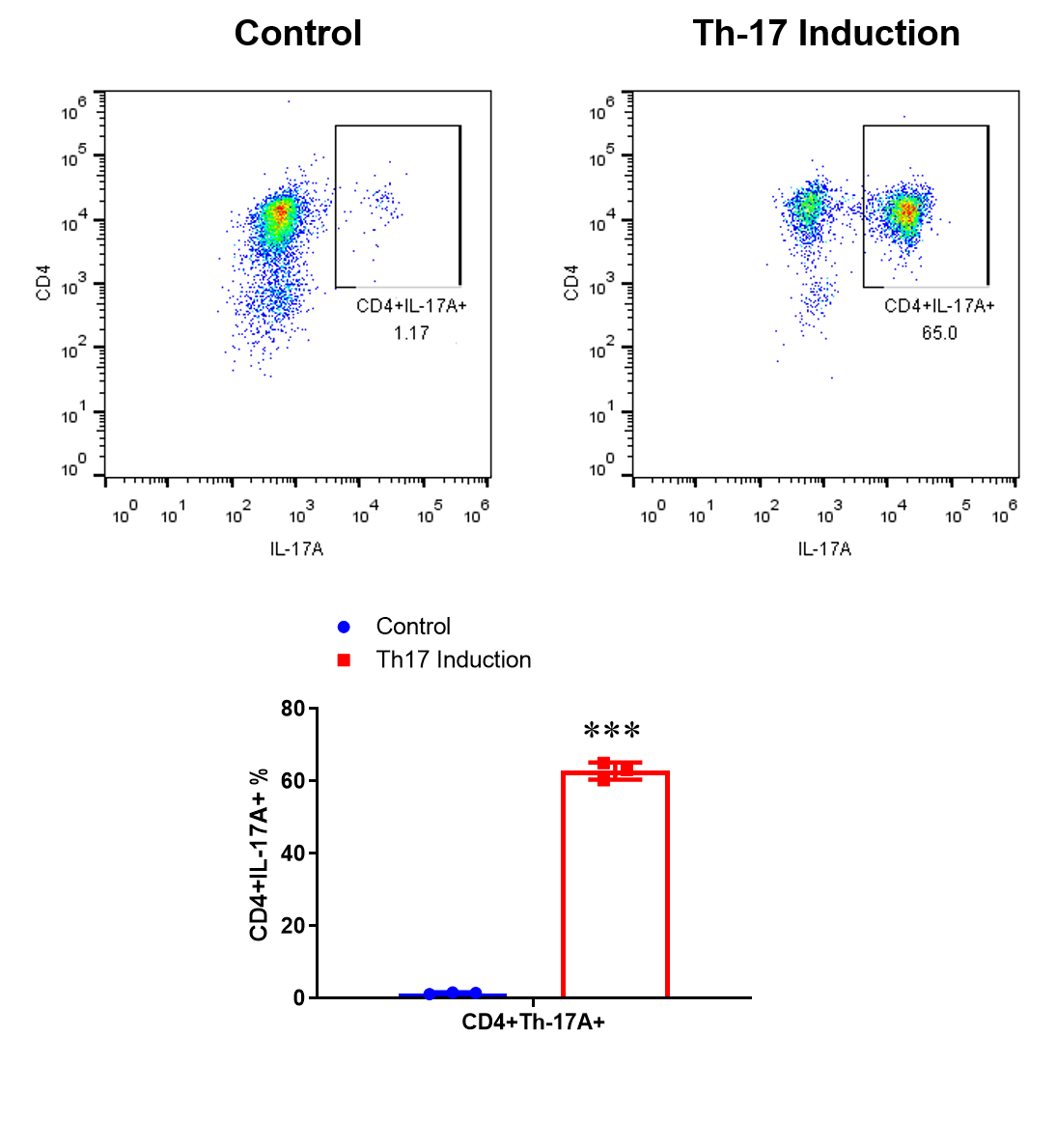


**Supplemental Figure 6.** Flow cytometry for Th17 cell differentiation from induced human CD4+ T cells. N=3.

**
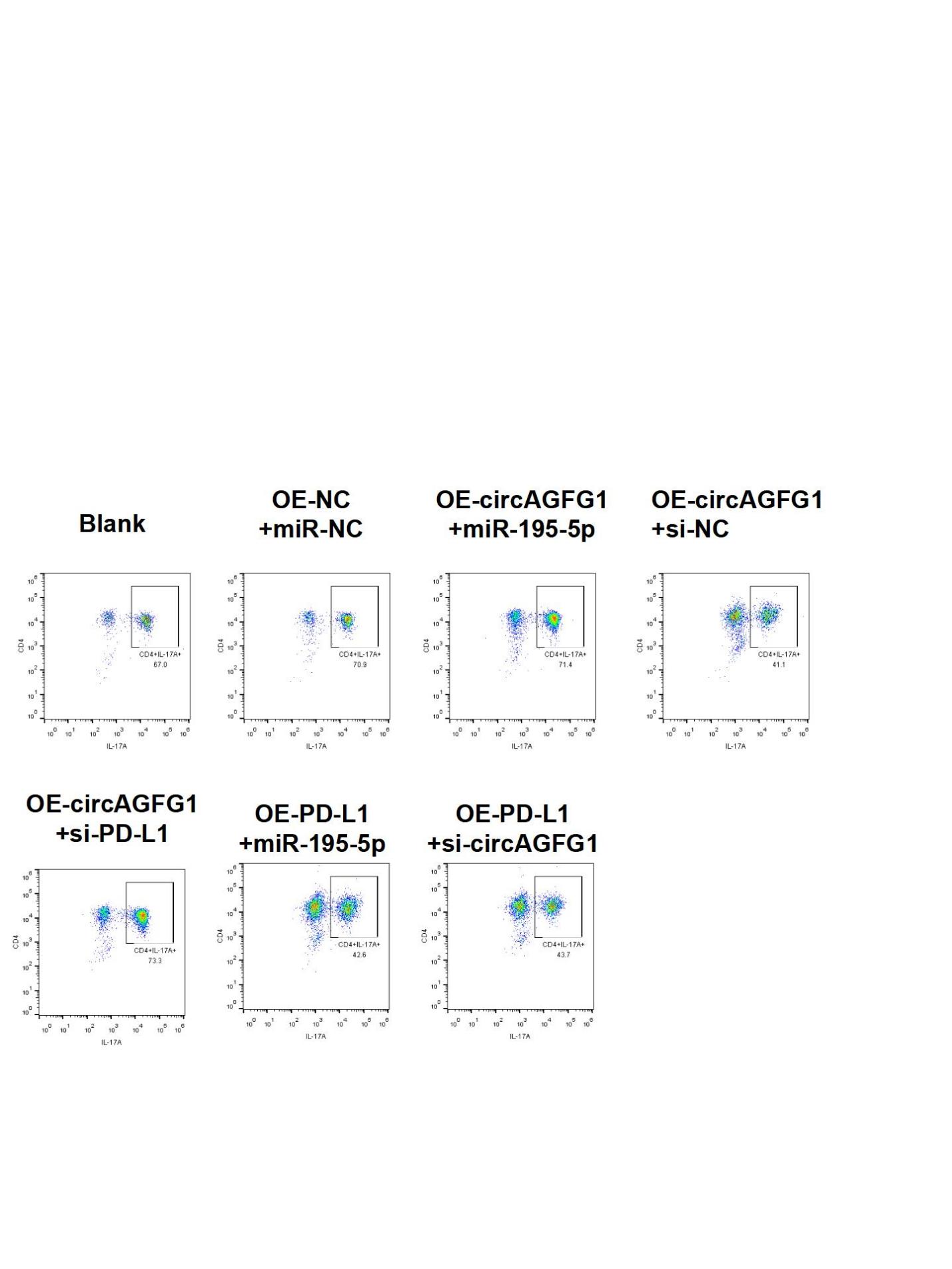
**

**Supplemental Figure 7.** Flow cytometry for Th17 cell differentiation when co-cultured with Calu-3 cells expressing the indicated construct(s). N=3.


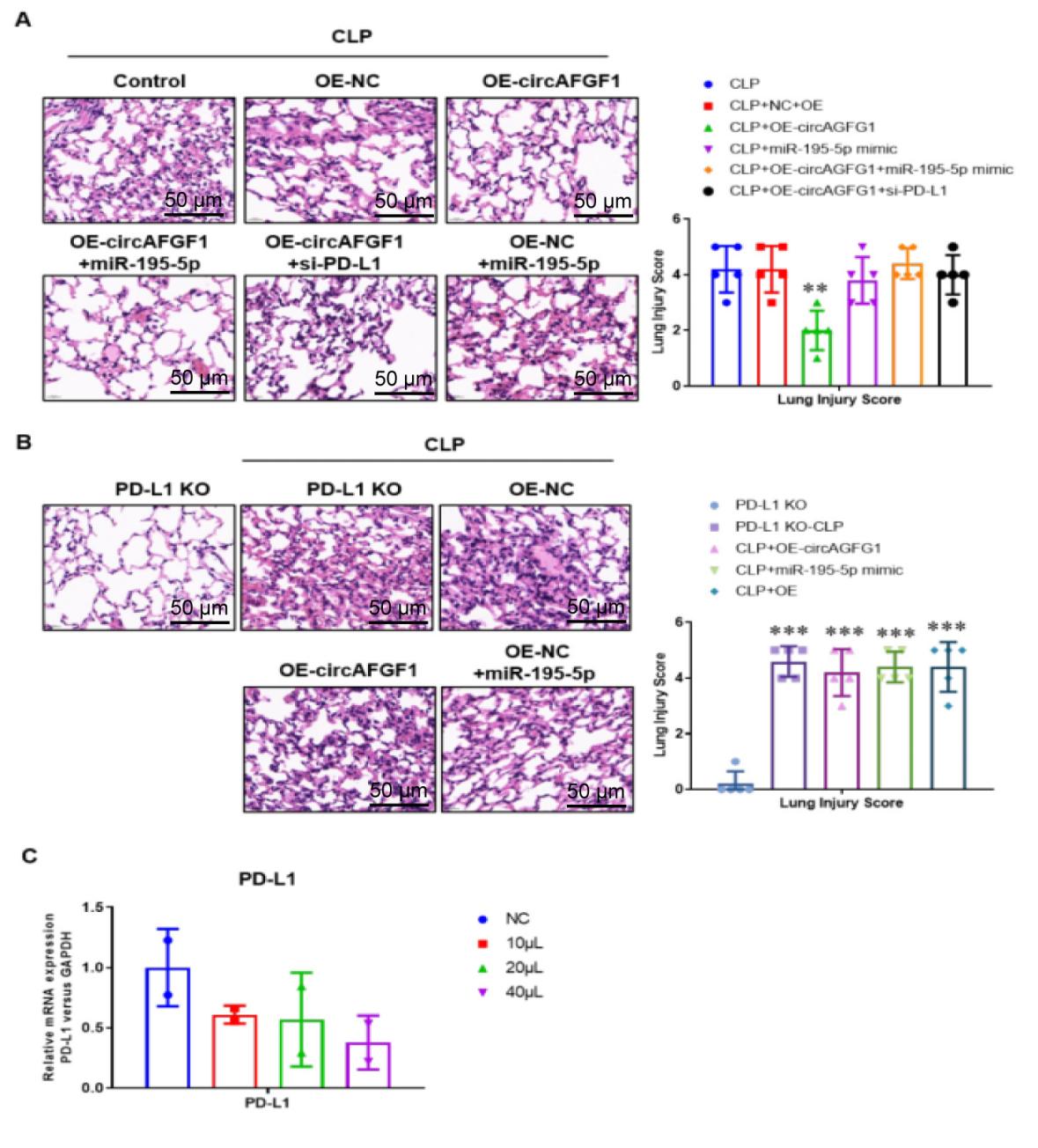


**Supplemental Figure 8.** A) HE staining of lung tissue from wild-type mice with cecal ligation and puncture (CLP)-induced sepsis locally injected with the indicated construct (s). B) HE staining of lung tissue from PD-L1 knockout (KO) mice with cecal ligation and puncture (CLP)-induced sepsis locally injected with the indicated construct (s). C) A PD-L1 construct was used to test the efficacy of local injection with different doses of construct. N=3, Bar = 50 µm.


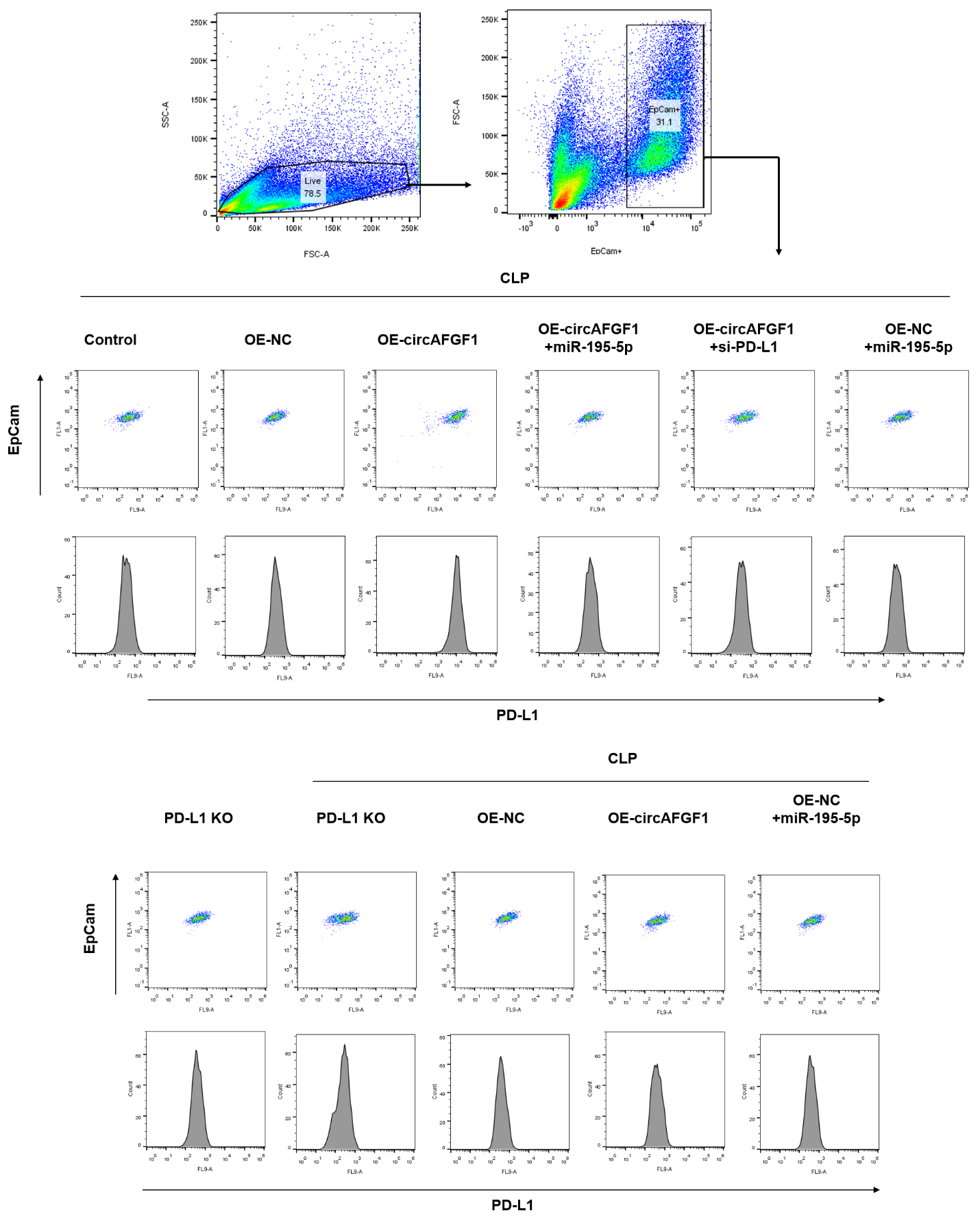


**Supplemental Figure 9.** Flow cytometry of PD-L1 expression in epithelial cells (EpCam) in lung tissue of wild-type (top) or PD-L1 knockout (KO, bottom) mice locally injected with the indicated construct(s). N=3.


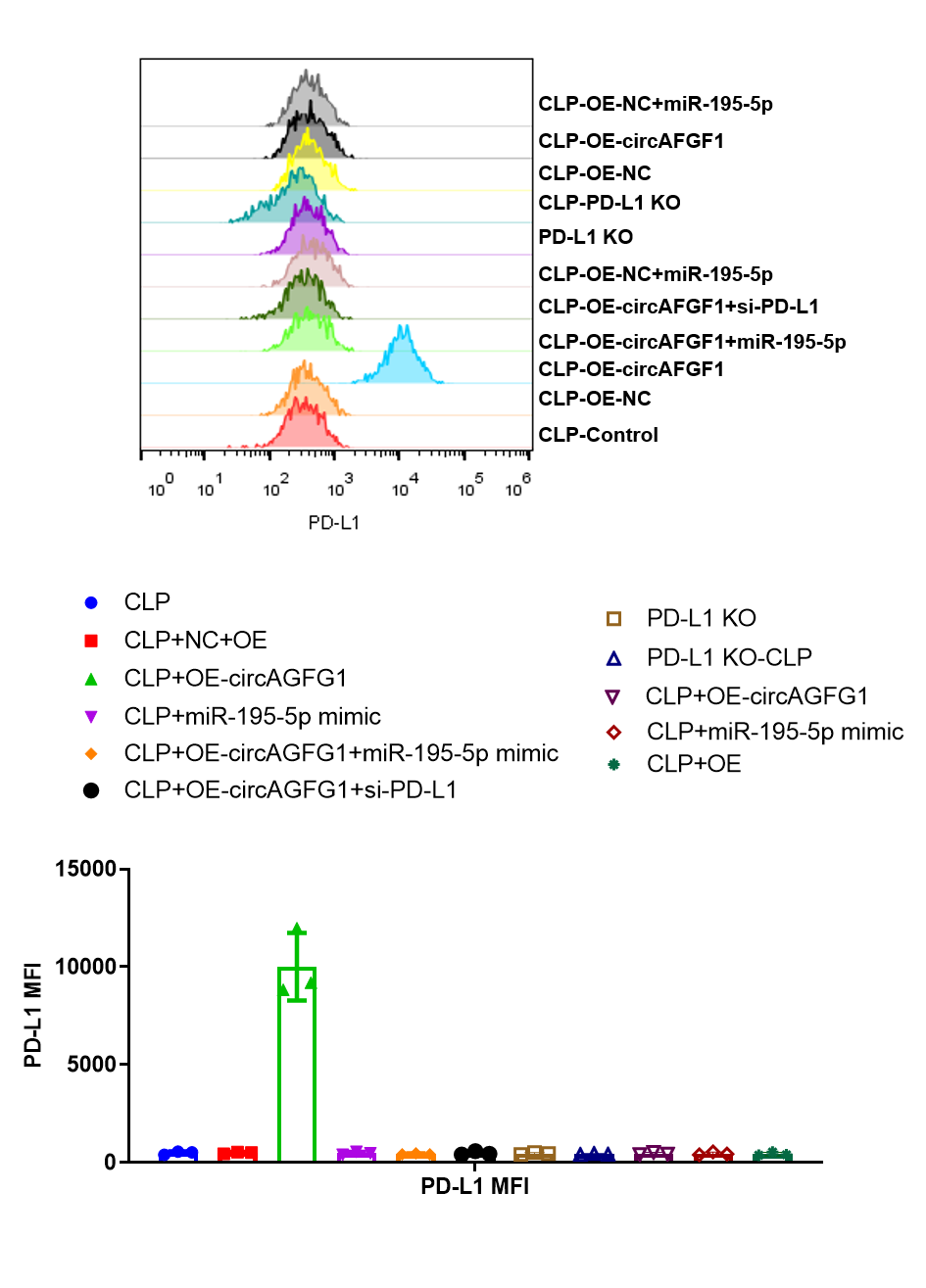


**Supplemental Figure 10.** Flow cytometry showing PD-L1 expression in lung tissue from wild-type or PD-L1 knockout (KO) mice with cecal ligation and puncture (CLP)-induced sepsis. N=3.


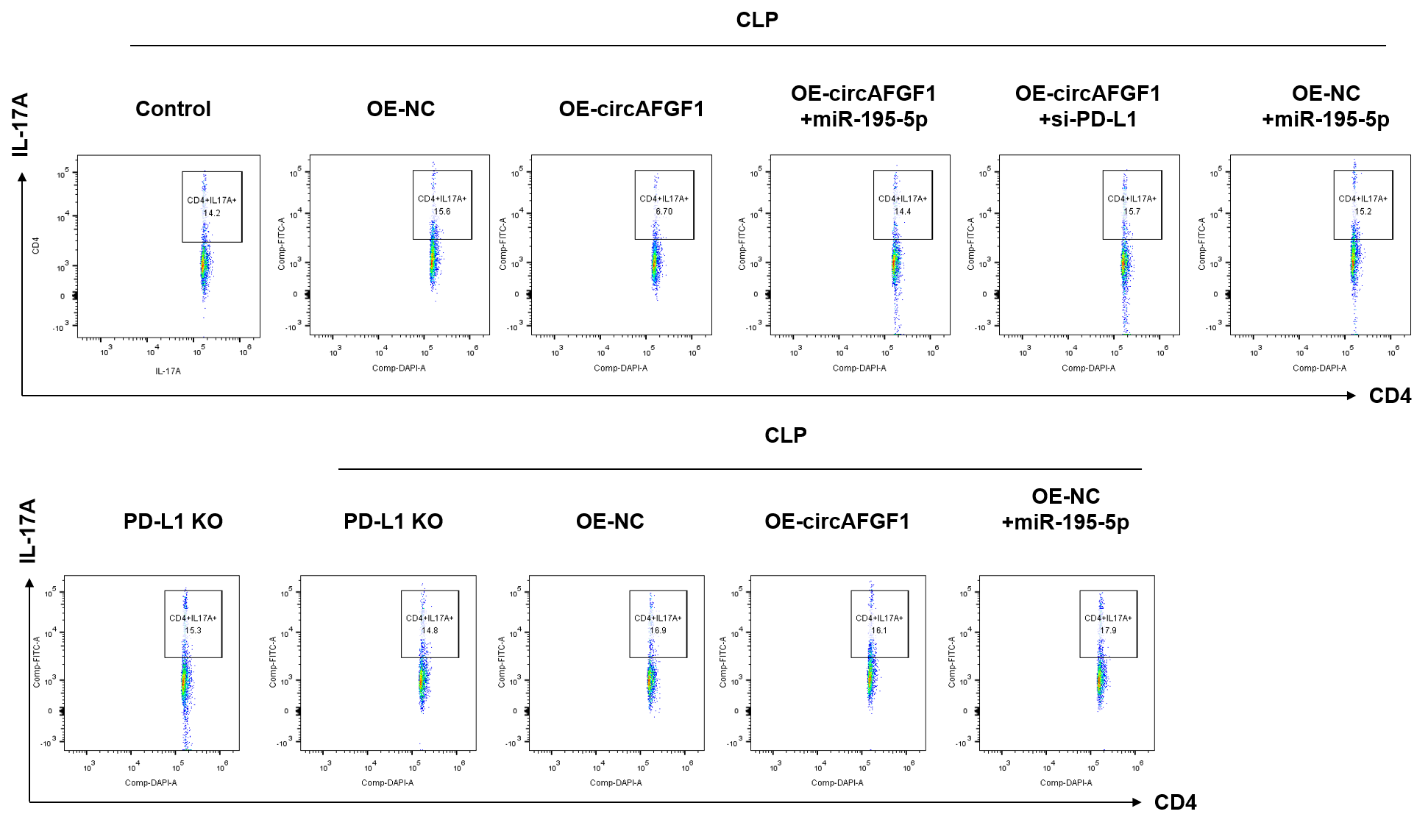


**Supplemental Figure 11.** Representative of flow cytometry results for CD4+ T cell IL-17 levels in lung tissue from wild-type (top) or PD-L1 knockout (KO, bottom) mice with cecal ligation and puncture (CLP)-induced sepsis. N=3.

**
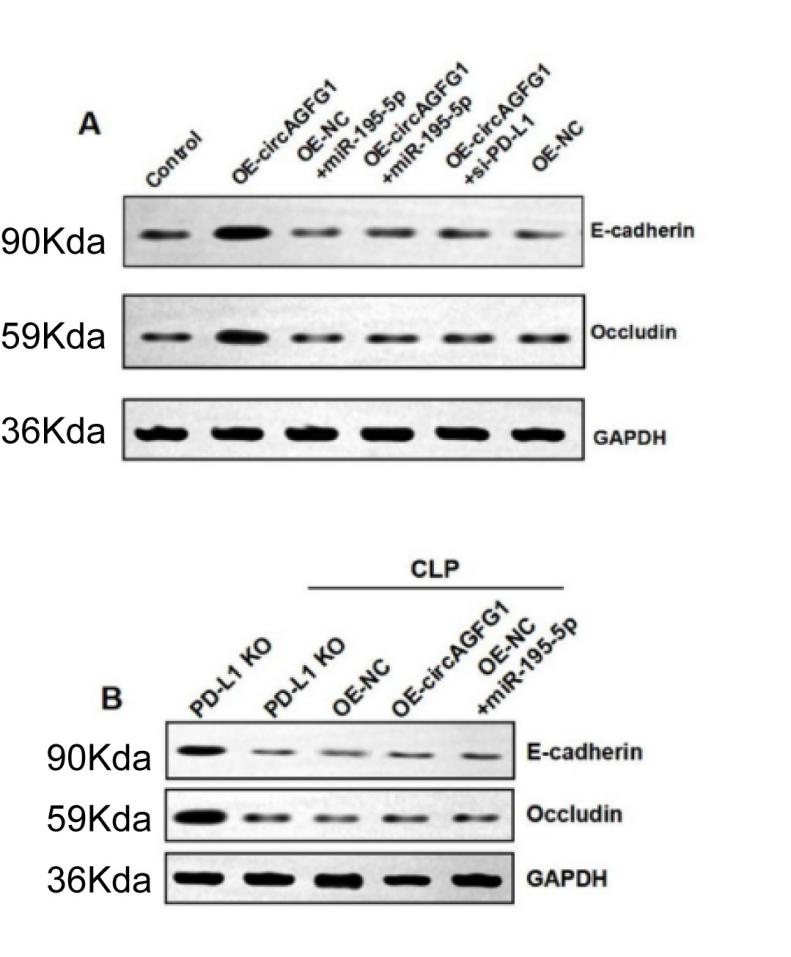
**

**Supplemental Figure 12.** Western blot replicates for E-cadherin, occludin, and GAPDH expression in lung tissue from A) wild-type and B) PD-L1 knockout (KO) mice with cecal ligation and puncture (CLP)-induced sepsis and expressing construct(s) injection as indicated. N=3.


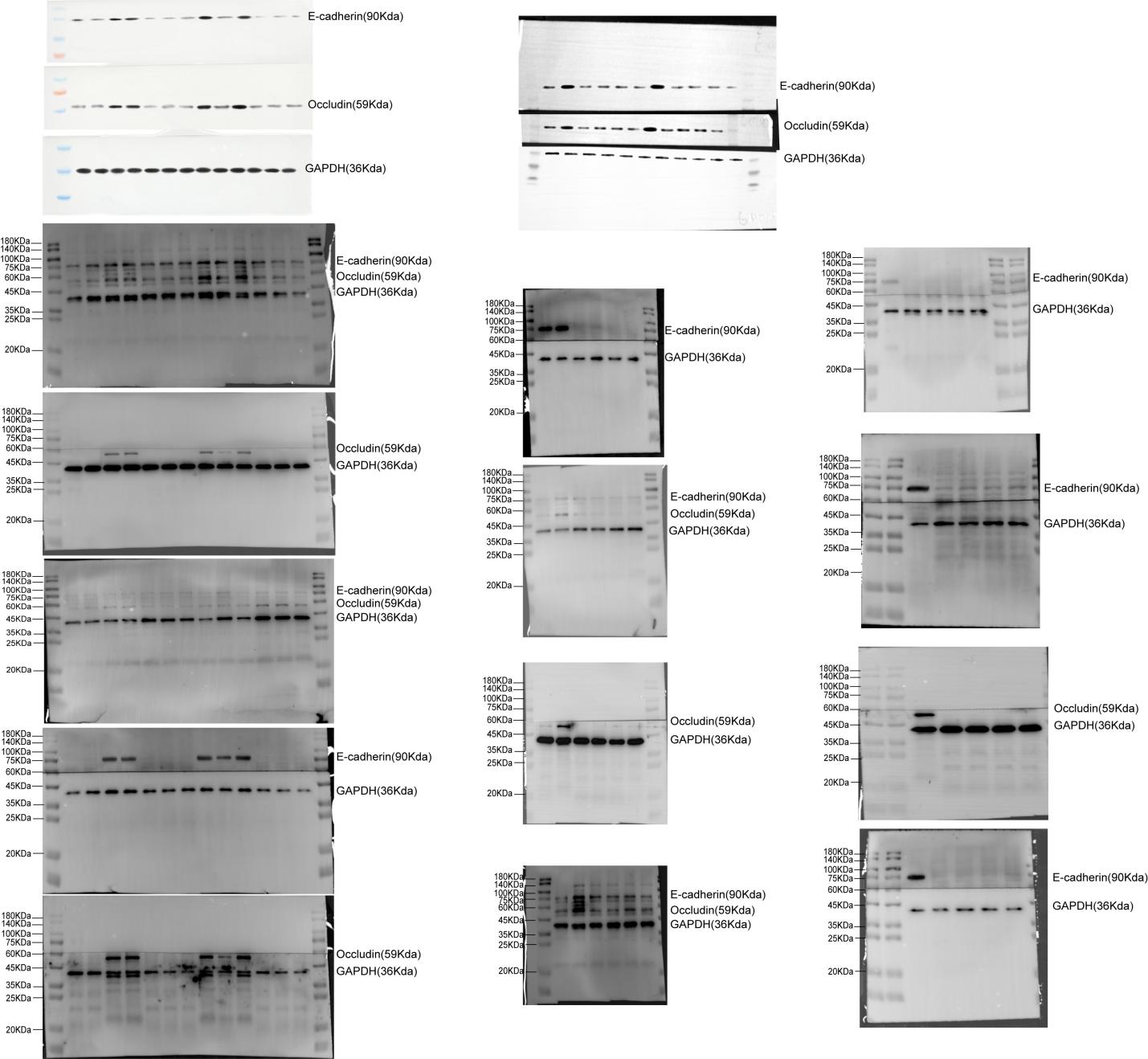


**Supplemental Figure 13.** Western blot raw data
